# Gut microbiota-derived 12-ketolithocholic acid suppresses the IL-17A secretion from colonic group 3 innate lymphoid cells to prevent the acute exacerbation of ulcerative colitis

**DOI:** 10.1080/19490976.2023.2290315

**Published:** 2023-12-08

**Authors:** Na Li, Peiguang Ma, Yalan Li, Xuekai Shang, Xinmei Nan, Lei Shi, Xiao Han, Jiajing Liu, Yanfei Hong, Qiuyi Li, Jiaqi Cui, Junxiang Li, Guiying Peng

**Affiliations:** a Department of Immunology and Microbiology, School of Life Sciences, Beijing University of Chinese Medicine, Beijing, People’s Republic of China; b Department of Gastroenterology, Dong Fang Hospital, Beijing University of Chinese Medicine, Beijing, People’s Republic of China

**Keywords:** Ulcerative colitis, secondary bile acids, gut microbiota, group 3 innate lymphoid cells

## Abstract

Intestinal microbiota dysbiosis and metabolic disruption are well-known as the primary triggers of ulcerative colitis (UC). However, their role in regulating the group 3 innate lymphoid cells (ILC3s), which are essential for intestinal health, remains unexplored during the development of disease severity. Here, our results showed that the microbiota structure of patients with severe UC (SUCs) differed from those with mild UC (MiUCs), moderate UC (MoUCs), and healthy controls (HCs). Microbes producing secondary bile acids (SBAs) and SBAs decreased with the aggravation of UC, and a strong positive correlation existed between them. Next, fecal microbiota transfer was used to reproduce the human-derived microbiota in mice and decipher the microbiota-mediated inflammatory modulation during an increase in disease severity. Mice receiving SUC-derived microbiota exhibited enhancive inflammation, a lowered percentage of ILC3s, and the down-regulated expressions of bile acid receptors, including vitamin D receptor (VDR) and pregnane X receptor (PXR), in the colon. Similar to clinical results, SBA-producing microbes, deoxycholic acids (DCA), and 12-ketolithocholic acids (12-KLCA) were diminished in the intestine of these recipients. Finally, we compared the therapeutic potential of DCA and 12-KLCA in preventing colitis and the regulatory mechanisms mediated by ILC3s. 12-KLCA but not DCA represented a strong anti-inflammatory effect associated with the higher expression of VDR and the lower secretion of IL-17A from colonic ILC3s. Collectively, these findings provide new signatures for monitoring the acute deterioration of UC by targeting gut microbiota and bile acid metabolism and demonstrate the therapeutic and preventive potential of a novel microbiota-derived metabolite, 12-KLCA.

## Introduction

Ulcerative colitis (UC) is a subtype of chronic inflammatory bowel disease (IBD) characterized by the recurrent inflammation of the colonic mucosal surface, which remains a continuously increasing health burden worldwide.^1^ The pathogenesis of UC remains incompletely understood but is thought to involve genetic, immune, and environmental threats, including disordered gut microbiota.^2^ Clinically, active UC is usually divided into mild, moderate, and severe subtypes based on the overall assessment of stool frequency, rectal bleeding, and colonoscopic activity.^3^ Typically, personalized medication is also provided for each category. Prominent microbiota imbalance has emerged as a significant inducement in the onset of UC.^2^ Several previous reports have also shown a significant association between gut microbiota shifts and disease severity.^4–8^ For instance, patients with the more severe disease exhibit lower bacterial community diversity,^4,6,8^ less bacterial members represented by Ruminococcaceae and Lachnospiraceae,^6^ but excessive *Escherichia* and *Shigella*.^8^

Besides, numerous microbial metabolites serve as crucial messengers to shape host immune homeostasis, especially bile acids (BAs) modified by bacteria-derived enzymes.^9–12^ Primary BAs (PBAs) are converted from cholesterol in the liver and secreted into the gut with taurine and glycine conjugates. Besides their role as emulsifiers, PBAs possess potent antimicrobial properties to modulate microbiota composition^13^. About 95% of PBAs are re-absorbed through the distal ileum and enter the enterohepatic circulation.^11^ The rest can be bio-transformed into secondary BAs (SBAs) by 7α-dehydroxylation in some members of Clostridial and Bacteroidaceae species to exert diverse metabolic effects and regulate innate and adaptive immunity.^11,14^ SBAs are well-known to reduce the host’s susceptibility to colitis through the regulation of colonic immune cells that express multiple bile acid receptors (BARs), such as vitamin D receptor (VDR), farnesoid X receptor (FXR), pregnane X receptor (PXR), G protein-coupled bile acid receptor 1 (GPBAR1), and liver X receptor (LXR).^11,12,15,16^ Notably, a lack of deoxycholic acids (DCA) and lithocholic acids (LCA) (usually the most abundant SBAs) and SBA-producing bacteria (e.g. Ruminococcaceae) have also been repeatedly reported in the intestine of patients with UC relative to controls.^17,18^ However, these studies have focused mainly on the difference between patients with pooled UC and controls, making it difficult to determine whether bile acid metabolism mediates the disease progression. The changes in bile acid profiles at different stages of UC require in-depth elucidation and exploration for potential personalized therapies.

Innate lymphoid cells (ILCs) contribute to host immunity, tissue homeostasis, and repair, which can be divided into three groups: group 1 ILCs (ILC1s) expressing T-bet, group 2 ILCs (ILC2s) expressing GATA3, and group 3 ILCs (ILC3s) expressing RORγt.^19^ The intestine is rich in ILC3, where they maintain homeostasis by orchestrating microbiota balance, barrier repair, host defense, and adaptive immunity. Commensal microorganisms in the intestine have a complex communication with ILC3s.^20^ ILC3s are the main producers of Th17-like (e.g. IL-17A) and Th22-like (e.g. IL-22) cytokines.^20,21^ IL-22 in the intestine accelerates epithelial repair and the production of antimicrobial peptides that limit intestinal inflammation.^22^ On the contrary, IL-17A has been shown to accelerate colonic injury via recruiting neutrophils to increase epithelial permeability in a review by Zeng et al.^21^ and our previous study.^23^ Bacterial metabolites, such as short-chain fatty acids (SCFAs), could maintain the function of group 3 innate lymphoid cells (ILC3s) by stimulating its differentiation, governing its receptor expression (e.g. VDR), and regulating the production of IL-17A and IL-22 in the presence of the RORγt.^20,21,24^ Two clinical investigations observed a lower level of colonic ILC3s in individuals with UC compared to controls.^2,25^ Nonetheless, a mechanistic understanding of the microbiota-SBAs-ILC3s associations in the deterioration of UC remains lacking.

Therefore, we hypothesized that microbial perturbation coupled with BAs metabolic instability would aggravate UC by regulating colonic ILC3s. In this study, we first conducted multi-center clinical recruitment in China involving a unique cohort of patients with mild (MiUCs), moderate (MoUCs), and severe UC (SUCs), as well as healthy controls (HCs) from 11 multiple clinical institutes. Microbial and metabolic characteristics among these patients with different severity of UC were compared based on the integrative analysis of 16S rRNA gene sequencing, untargeted and targeted metabolomics, and machine learning algorithms. Next, human-associated fecal microbiota transplantation (FMT) was conducted using an antibiotic-treated mouse model, in combination with flow cytometry, to validate the ILC3s-associated inflammatory modulation mediated by human-derived intestinal microbiota and SBAs during the increase in disease severity. Finally, a DSS-induced colitis mouse model and ILC3s sorting *in vitro* were used to explore the role of DCA and 12-ketolithocholic acids (12-KLCA), two SBAs less abundant in mice receiving SUCs-derived FMT, in the regulation of colonic inflammation and ILC3s function. We identified one specific microbial metabolite (12-KLCA) that exerts a potent anti-inflammatory effect by inhibiting the secretion of IL-17A from colonic ILC3s. Our findings would promote the exploration of new approaches to the prevention and treatment of the acute exacerbation of UC by targeting the gut microbiota and bile acid metabolism.

## Results

### The microbiota structure of SUCs differs from that of MiUCs and MoUCs

**Figure 1.**
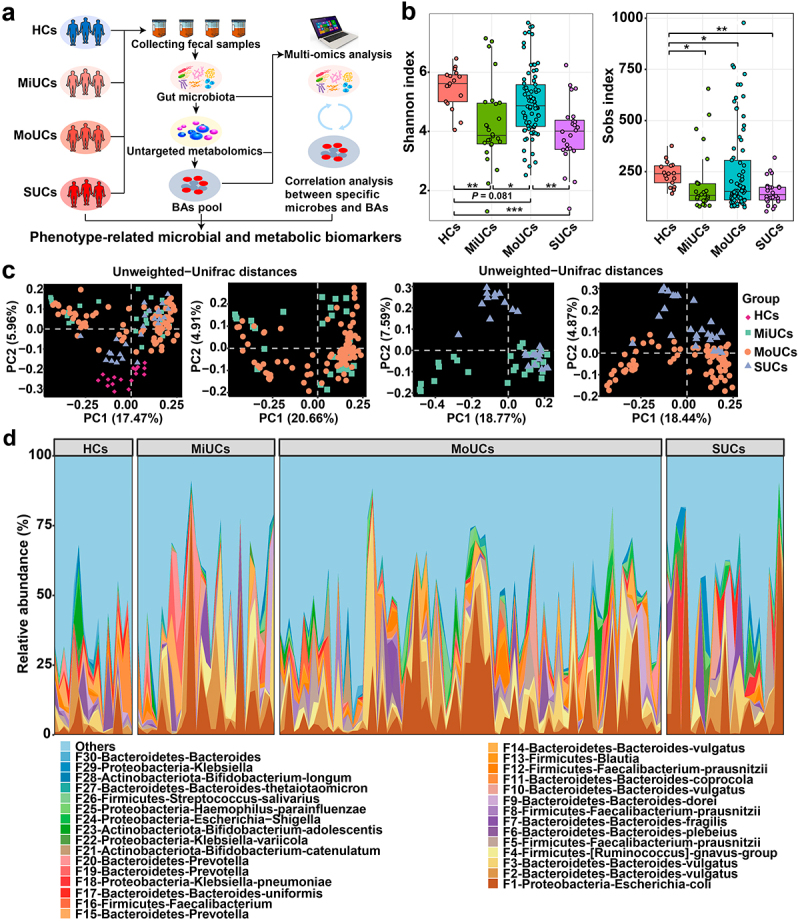
Overall gut microbiota structure of SUCs differed from that of MiUCs and MoUCs. (a) fecal samples from all human subjects were collected for microbiome and metabolomics analysis. (b) alpha-diversities of gut microbiota profiles calculated using the Shannon and Sobs indexes; Statistical differences were calculated by the Kruskal-Wallis test with a false discovery ate (FDR) correction. ***P < 0.001, **P < 0.01, and *P < 0.05. (c) beta-diversities of gut microbiota profiles illustrated with PCoA using Unweighted-Unifrac matrix. (d) relative abundances of the top 30 bacterial features. HCs (*n* = 17), MiUCs (*n* = 29), MoUCs (*n* = 79), and SUCs (*n* = 25).

We first examined if the microbiota composition of volunteers with mild, moderate, and severe UC followed a severity-specific pattern in the clinical cohorts (Figure 1a). All UC patients had a dramatically lower Shannon index and observed bacterial features compared to controls (Figure 1b), indicating poorer microbial richness and evenness in these UC subjects. Principal coordinates analysis (PCoA) coupled with permutational multivariate analysis of variance (PERMANOVA) suggested that UC samples clustered separately from HCs, based on Unweighted-Unifrac and Jaccard distances (*P* < 0.01; Figure 1c, Supplementary Table S1, and Supplementary Figure S1a). In particular, pair-wise comparisons further revealed that the community structure of SUCs significantly differed from that of MiUCs and MoUCs (Unweighted-Unifrac, *P* < 0.10; Jaccard, *P* < 0.05). However, no difference was found between MiUCs and MoUCs (Unweighted-Unifrac, *P* = 0.51; Jaccard, *P* = 0.11). Besides, PERMANOVA showed no significant difference between female and male subjects (Unweighted-Unifrac, *P* = 0.51; Jaccard, *P* = 0.75), so the gender effect was not considered in the downstream analysis. Moreover, the community composition analysis also uncovered significant shifts in the microbial consortia among different groups (Figure 1d, Supplementary Figure S1b and 1c, and Supplementary Figure S2). The predominant phylum Bacteroidetes was less abundant in MoUCs and SUCs than that in HCs. Notably, all UC samples exhibited higher proportions of the phylum Proteobacteria and the genus *Escherichia-Shigella* compared to HCs, with the highest value in SUCs (Supplementary Figure S2). These observations implicated that the overall microbiota structure in SUCs was markedly distinguished from MiUCs and MoUCs.

### Microbial indicators associated with disease severity

**Figure 2.**
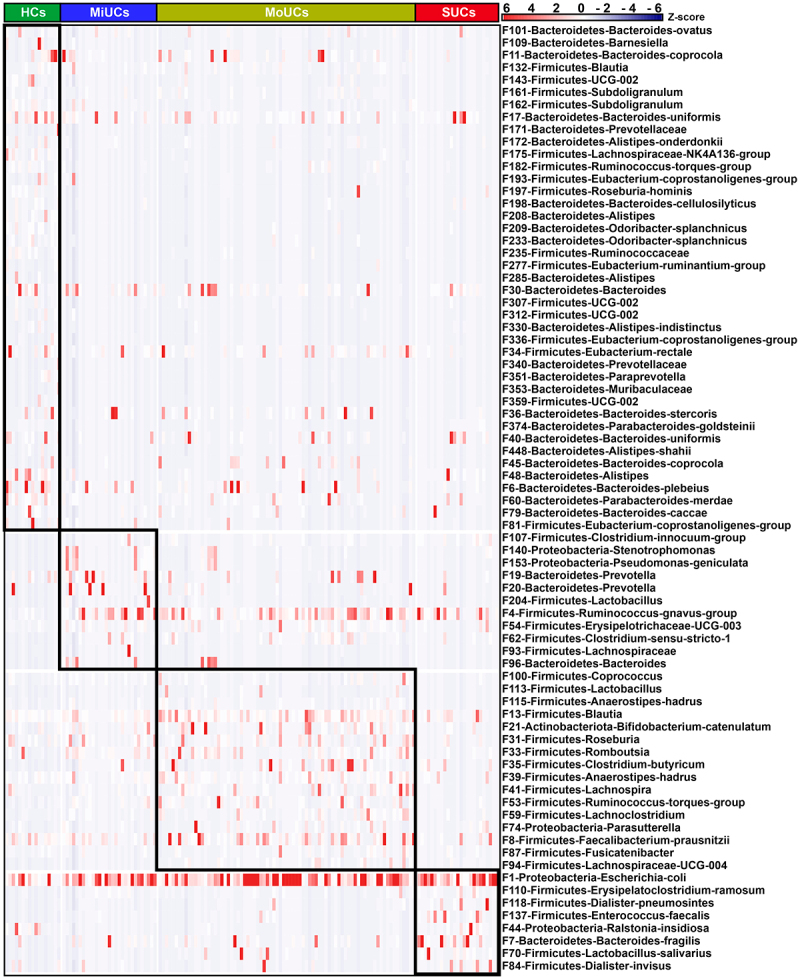
Heatmap showing 76 disease activity-associated bacterial features identified by LEfSe. The top 500 features were used for LEfSe analysis (LDA >2). Cells are colored based on a Z-score scale of bacterial abundances. HCs (*n* = 17), MiUCs (*n* = 29), MoUCs (*n* = 79), and SUCs (*n* = 25).

Next, we sought to identify the disease activity-associated bacterial features using linear discriminant analysis (LDA) effect size (LEfSe) based on the top 500 features, an algorithm focusing on both statistical significance and biological consistency. As shown in Figure 2, LEfSe analysis indicated 11, 16, and 8 features within the phyla Firmicutes, Bacteroidetes, Proteobacteria, and Actinobacteria, were specifically enriched in MiUCs, MoUCs, and SUCs, respectively. Among them, F140 (*Stenotrophomonas*) and F153 (*Pseudomonas-geniculata*) in MiUCs, F74 (*Parasutterella*) in MoUCs, and F44 (*Ralstonia-insidiosa*) in SUCs exhibited the higher abundances, and they were assigned to Proteobacteria. Interestingly, F1 (*Escherichia coli*), belonging to the genus *Escherichia-Shigella* in SUCs, was strongly enriched in SUCs, which suggested that F1 might be a pivotal exacerbator mediating the development of SUCs. In contrast, a total of 41 features, such as F175 (*Lachnospiraceae-NK4A136-group*), F182 (*Ruminococcus-torques-group*), F235 (Ruminococcaceae), F336 (*Eubacterium-coprostanoligenes-group*), and F81 (*Eubacterium-coprostanoligenes-group*), were much less abundant in MiUCs, MoUCs, and SUCs than those in HCs.

**Figure 3.**
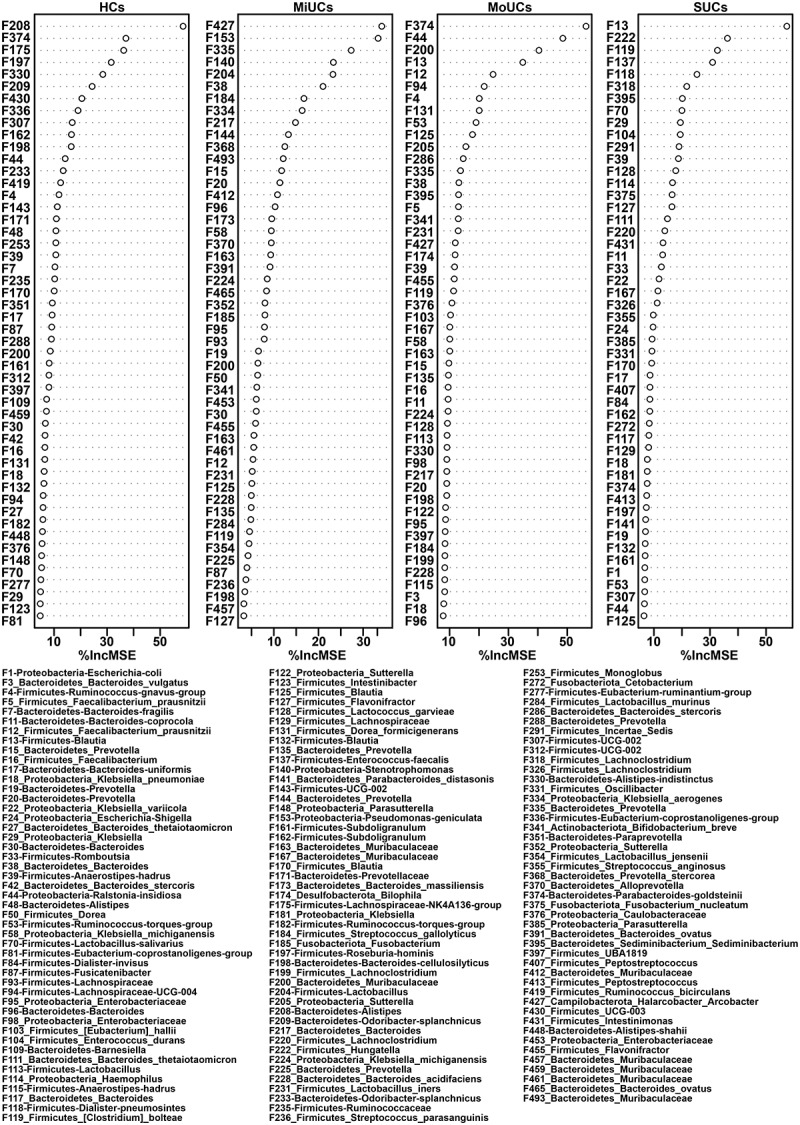
Random forest revealing disease activity-related features at each type of UC. The top 50 disease activity-related bacteria of HCs, MiUCs, MoUCs, and SUCs were selected from the top 500 features using a regression-based random forest algorithm in R. HCs (*n* = 17), MiUCs (*n* = 29), MoUCs (*n* = 79), and SUCs (*n* = 25).

We subsequently performed the regression-based random forest by using the disease severity as the consequence and the top 500 features as predictors. The top 50 features used to predict disease severity are listed in Figure 3. Consistently, these features include most of the observations mentioned in Figure 2. For example, F175, F182, F235, F336, and F81, less abundant in UC samples, were again listed as HC-related features. F153, together with F140, was again confirmed to be positively related to MiUCs. Additionally, F113 (*Lactobacillus*) and F115 (*Anaerostipes-hadrus*), as well as F1 was positively associated with MoUCs and SUCs, respectively. Moreover, the shared indicators of LEfSe and random forest focused on 48 bacterial features (Supplementary Figure S3). Remarkably, most HC-related features displayed the lowest abundance in SUCs compared with other patients, especially F11 (*Bacteroides-coprocola*), F132 (*Blautia*), F143 (*UCG-002*), F161 (*Subdoligranulum*), F182, F235, and F374 (*Bacteroides-uniformis*) (Supplementary Figure S3).

Finally, network analysis using the SPIEC-EASI algorithm further showed dramatic interactions among most of these shared features (Supplementary Figure S4). Surprisingly, 7 clusters with a total of 39 features were observed in the network, which constituted a complete ecological network connected with each other. The connected nodes (bacterial features) in each cluster presented associations with 2 to 3 types of disease status. Notably, purple and yellow clusters displayed a partially overlapped region consisting of four features, such as F81, F109 (*Barnesiella*), and F312 (*UCG-002*), which further connected the remaining clusters including F1, F175, F182, and F336.

Collectively, these features identified by disease status, exhibiting synergistic changes and close ecological interactions, might serve as critical microbial indicators co-mediating the transition from MiUCs to MoUCs and then to SUCs.

### The intestinal biosynthesis of SBAs is deficient in UC patients

**Figure 4.**
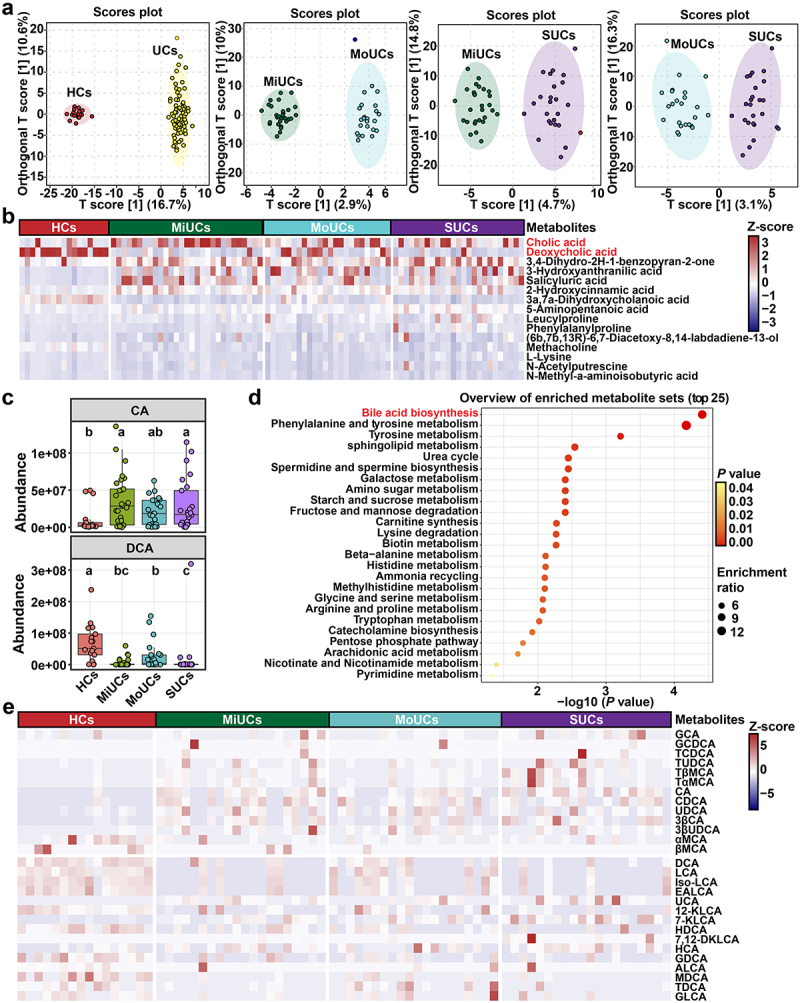
Multiple metabolomics showing significant differences in BAs production and biosynthesis among different groups. (a) OPLS-DA plots of fecal metabolites obtained by untargeted metabolomics; the robust separation based on OPLS-DA analysis indicates the significant discrimination in metabolic profiles between groups. (b) heatmap showing the top 15 differentially abundant metabolites identified by untargeted metabolomics using multiple Mann-Whitney *U*-tests with FDR correction between every two groups. (c) boxplots showing BAs obtained by untargeted metabolomics that accumulate or decrease with the increase of disease activity; Statistical differences were calculated by Kruskal-Wallis test with FDR correction; ^a, b, c^ different letters within each panel represent a significant difference (*P* < 0.05). (d) metabolic pathway enrichment analysis of differentially abundant metabolites obtained by untargeted metabolomics based on the KEGG database. HCs (*n* = 17), MiUCs (*n* = 29), MoUCs (*n* = 24), and SUCs (*n* = 25). (e) differentially abundant PBAs and SBAs obtained by targeted metabolomics; cells in heatmaps are colored based on a Z-score scale of metabolite abundances. HCs (*n* = 16), MiUCs (*n* = 20), MoUCs (*n* = 20), and SUCs (*n* = 20).

To assess microbial metabolic fluctuations in patients with UC, we undertook untargeted fecal metabolomics to roughly profile the potential metabolites associated with the disease severity of UC. Orthogonal partial least squares discriminant analysis (OPLS-DA) revealed significant segregation between every two groups of participants (Figure 4a). A total of 477 differentially abundant metabolites were identified, among which the top 15 compounds are shown in Figure 4b. Typically, cholic acids (CA) and DCA were the two most abundant differential metabolites. Besides, compared to HCs, a higher content of CA was observed in MiUCs and SUCs but a lower abundance of DCA in MiUCs, MoUCs, and SUCs (Figure 4b,c). Metabolic pathway enrichment analysis further indicated that bile acid biosynthesis was the most critical pathway mediated by these differentially abundant metabolites (Figure 4d).

Based on the above-mentioned results, we further performed targeted metabolomics and found a total of 28 BAs changed with the increase in disease severity (Figure 4e and Supplementary Figure S5). For example, the dominant conjugated PBAs, including glycocholic acids (GCA) and taurocholic acids (TCA), were the most abundant in SUCs than those in HCs, MiUCs, and MoUCs. Several PBAs were simultaneously enriched in different degrees of UC, with the highest abundance in SUCs. These PBAs mainly comprised CA, chenodeoxycholic acids (CDCA), ursodeoxycholic acids (UDCA), and 3β-cholic acids (3β-CA). By contrast, SBAs, including DCA and LCA converted from CA and CDCA by specific microbes, respectively, exhibited lower abundances in the intestine of UC patients compared to HCs. Consistently, an array of SBA derivatives were also less abundant in UC patients than HCs, such as isolithocholic acids (iso-LCA), epiallolithocholic acids (EALCA), 12-KLCA, and hyodeoxycholic acids (HDCA). Strikingly, almost all of these SBAs presented the smallest value in SUCs relative to HCs, MiUCs, and MoUCs.

These results proved that the intensification of UC was accompanied by the gradual accumulation of PBAs and the decrease of SBAs. UC-derived microbiota might have a poor ability to transform PBAs into SBAs, thereby sharpening colonic inflammation to promote the condition aggravation.

### Disease-associated features interact with deficient SBAs as potential diagnostic indicators for UC

**Figure 5.**
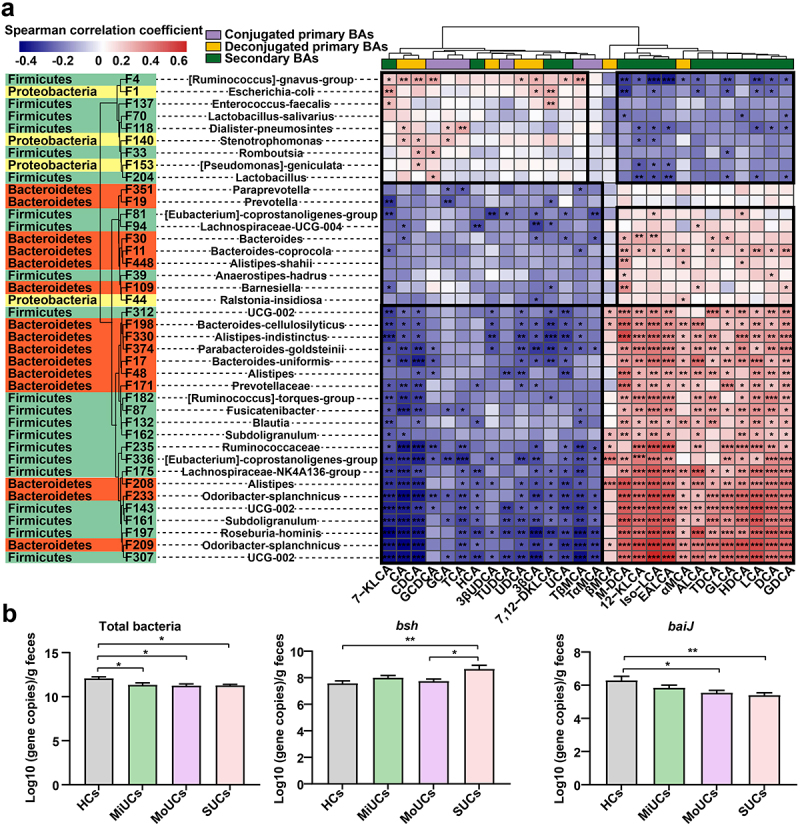
Spearman’s correlation and absolute qPCR revealing the relationship between BA-producing microbes and the BAs pool. (a) correlation matrix between microbes and metabolites affected by the disease severity of UC; cells are colored based on the Spearman correlation coefficient between candidate bacterial features and metabolites; *P* values less than 0.05 were considered a significant correlation; the red represents a positive correlation, the blue represents a negative correlation, and the white represents no significant correlation. (b) the copy number of total bacteria, *bsh*, and *baiJ* genes of HCs (*n* = 16), MiUCs (*n* = 16), MoUCs (*n* = 18), and SUCs (*n* = 18); Statistical differences were calculated by one-way ANOVA with Tukey’s *post hoc* test; ***P < 0.001, **P < 0.01, and *P < 0.05.

A Spearman’s correlation matrix was subsequently generated to explore the interactions between bacterial features and candidate BAs that altered with the disease activity (Figure 5a). Most UC-related bacterial members presented significant associations with the altered BAs pool. A small cluster of features was positively correlated to abundant PBAs (e.g. CA, CDCA, GCA, TCA, UDCA, and 3β-CA) but negatively connected with deficient SBAs (e.g. 12-KLCA, iso-LCA, EALCA, HDCA, LCA, and DCA). These features included F4 (*[Ruminococcus]-gnavus-group*), F1, F118 (*Dialister-pneumosintes*), F140 (*Stenotrophomonas*), F33 (*Romboutsia*), F153 (*[Pseudomonas]-geniculata*), and three members (e.g. F137 (*Enterococcus-faecalis*), F70 (*Lactobacillus-salivarius*), and F204 (*Lactobacillus*)) within Lactobacillales, all of which were enriched in UC patients compared with HCs. Certain microbial members, like Lactobacillus and *Escherichia coli*, encode the BA hydrolase (*bsh*) gene to realize the deconjugation of PBAs.^11,13^ The qPCR quantification test further confirmed that the copy numbers of microbial *bsh* genes were significantly higher in SUCs than HCs and MoUCs (Figure 5b). These suggested that the accumulation of dominant PBAs might be caused by the enrichment of these above PBA-producing features in this study.

On the other hand, most members of Clostridial and Bacteroidaceae can transform PBAs into SBAs through the 7α/β-dehydroxylation due to harboring the bile acid-inducible (*baiJ*) gene.^14^ Interestingly, a complicated subset of features within these two microbial divisions, less abundant in UC patients than HCs, showed strongly inverse relationships with PBAs but positive correlations with SBAs mentioned above (Figure 5a). These microbes mainly contained F81, F94 (*Lachnospiraceae-UCG-004*), F11, F109, F312, F374, F182, F132, F235, F336, F175, F208 (*Alistipes*), F143, and F197 (*Roseburia-hominis*), *etc* (Figure 5a). Meanwhile, the qPCR results showed that SUCs possessed the less *baiJ* genes than HCs and MoUCs (Figure 5b). In other words, the deficiency of SBA-producing microorganisms might induce the inadequate production of SBAs during the aggravation of UC.

We again undertook logistic regression models with 10-fold cross-validation to investigate the potential of these interacting metabolites and bacterial markers for noninvasive diagnosis across the distinct stages of UC. As shown in Supplementary Figure S6a and 6b, the combination of 21 BAs and 40 bacterial features as markers achieved a high area under the curve (AUC) of 0.84 and 0.88 in MiUCs and MoUCs, respectively. Importantly, these above 61 signatures discriminated SUCs from non-SUCs, with a much higher AUC of 0.94 (Supplementary Figure S6c), again demonstrating their great credibility as potential diagnostic indicators co-mediating the deterioration of UC.

### Gut microbiota from SUCs induces colonic inflammation in recipients

**Figure 6.**
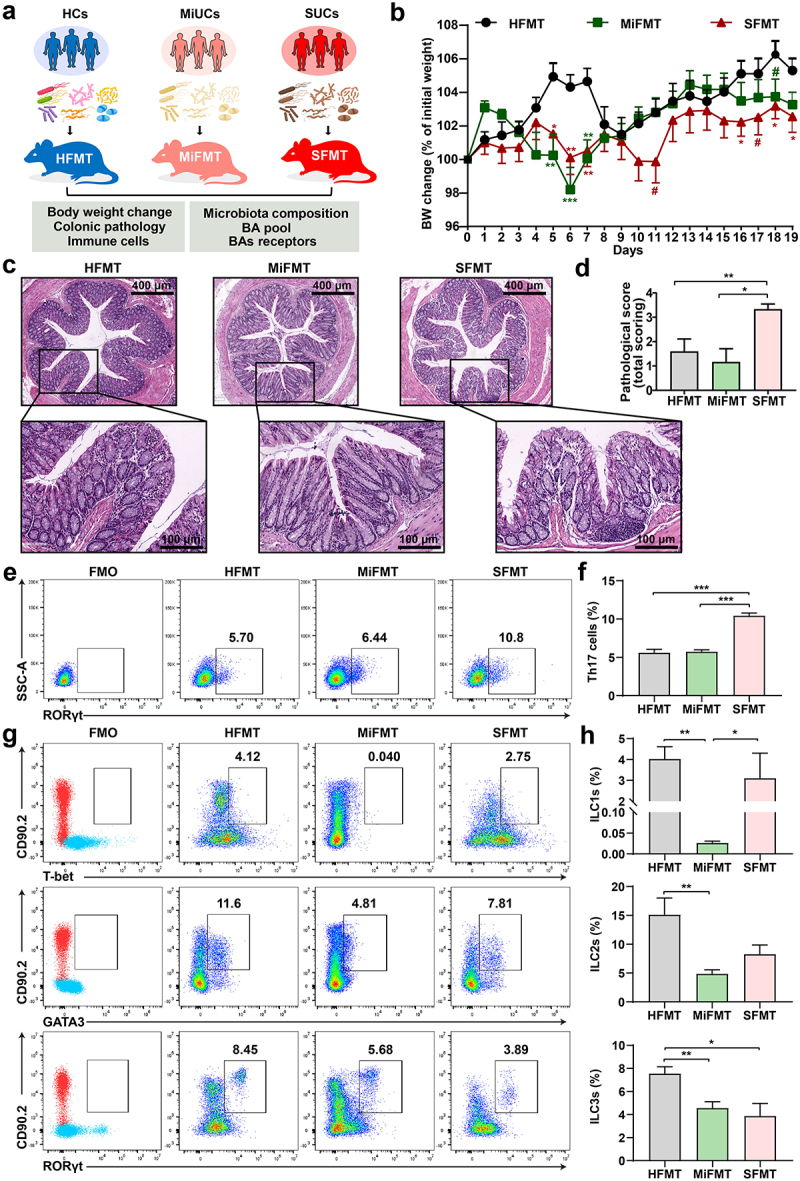
Gut microbiota from SUCs induced colonic inflammation and lowered colonic Th17 and ILC3 in recipients. (a) the protocol of animal treatments and sample collection. (b) body weight change of mice throughout the entire trial; HFMT (*n* = 12), MiFMT (*n* = 6), and SFMT (*n* = 12). (c) H&E staining of the colorectum. (d) a summarized histopathological score of the colorectum; HFMT (*n* = 5), MiFMT (*n* = 6), and SFMT (*n* = 6). (e) Representative scatter plots for identifying Th17 cells in the colon quantified by flow cytometry; Th17 cells were identified as CD3^+^CD4^+^RORγt^+^cells. (f) the percentages (% CD3^+^CD4^+^cells) of Th17 cells; *n* = 5 per group. (g) Representative scatter plots for identifying ILC1s, ILC2s, and ILC3s in the colon; ILC1s were identified as CD45^+^Lin^−^CD90.2^+^T-bet^+^cells, ILC2s as CD45^+^Lin^−^CD90.2^+^GATA3^+^cells, and ILC3s as CD45^+^Lin^−^CD90.2^+^RORγt^+^cells. (h) the percentages (% CD45^+^cells) of ILC1s, ILC2s, and ILC3s. *n* = 5 per group. Statistical differences (b, d, f, and h) were calculated by one-way ANOVA with Tukey’s *post hoc* test. ***P < 0.001, **P < 0.01, *P < 0.05, and ^#^*P* < 0.10.

We hypothesized that the alterations in SBAs-producing microbes of these patients represent a mechanism by which the deficiency of SBAs promotes the exacerbation of colonic inflammation. To test this, we first colonized adult antibiotic-treated mice with fecal microbiota from MiUCs, SUCs, and HCs, respectively (Figure 6a). Three groups of recipients were generated including mice receiving HC-associated FMT (HFMT), MiUC-associated FMT (MiFMT), and SUC-associated FMT (SFMT). As shown in Supplementary Figure S7, the antibiotic treatment (ABT) induced a pronounced decrease in the copies of total bacteria and alpha diversity of mice, indicating an ideal depletion of murine endogenous intestinal microbiota.

Compared with HFMT, both MiFMT and SFMT significantly reduced the body weight of their recipients to various degrees (Figure 6b). Notably, mice with SFMT exhibited a greater and more continuous body weight loss than MiFMT. Furthermore, SFMT induced excessive pro-inflammatory cell infiltration, crypt damage, disordered goblet cells, and therefore higher histopathological scores in the colorectum of recipients than HFMT and MiFMT (Figure 6c,d). However, no noticeable histology change was observed between HFMT and MiFMT. These observations suggested that only SFMT caused a phanerous colonic inflammatory tone in antibiotic-treated mice.

### Gut microbiota from SUCs decreases the population of colonic Th17 and ILC3s in recipients

Numerous studies have demonstrated the vital role of Th17 cells that could be regulated by microbial SBAs in exacerbating colitis in mice.^12,26,27^ Hence, we next addressed whether the FMT from different degrees of UC induced different differentiation patterns of Th17 cells in their recipients (Figure 6e,f). As expected, a remarkable accumulation of RORγt^+^Th17 cells was seen in the colon of mice with SFMT compared to HFMT and MiFMT. Given that intestinal ILC3s are well-known as the mirror cells of Th17 cells on account of the property to produce IL-17.^21^ We subsequently explored whether MiFMT and SFMT induced the abnormity of ILC3s by examining ILC1s, ILC2s, and ILC3s (Figure 6g,h). We found that SFMT had no significant impact on ILC1s and ILC2s compared to HFMT, although MiFMT induced a decrease in ILC1s and ILC2s. However, only the population of ILC3s exhibited a synchronous decrease after SFMT and MiFMT relative to HFMT, with SFMT having the lowest value, implicating the priority of ILC3s in orchestrating the onset and exacerbation of colonic inflammation.

### Gut microbiota from SUCs diminishes 12-KLCA positively correlated with the shortage of ILC3s in recipients

**Figure 7.**
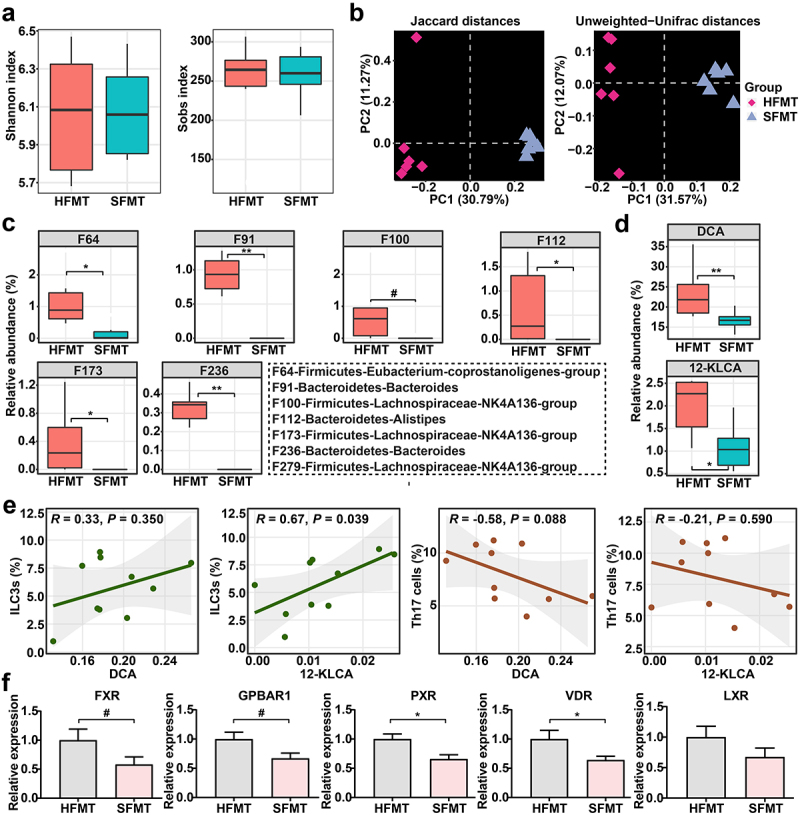
Gut microbiota from SUCs reduced the relative abundances of SBA-producing microbes and SBAs in recipients. (a) alpha-diversity of gut microbiota profiles, calculated using the Shannon and Sobs indexes. (b) beta-diversity of gut microbiota profiles illustrated with PCoA using Jaccard and Unweighted-Unifrac distances. (c) SBA-producing bacterial features that were absent in clinical SUCs and mice with SFMT. (d) SBAs that were absent in clinical SUCs and mice with SFMT. (e) associations between key SBAs, ILC3s, and Th17 cells affected by SFMT; correlation coefficients and statistical differences were calculated by the Spearman correlation pipeline; *P* values less than 0.05 were considered a significant correlation. (f) the mRNA expressions of BARs including FXR, GPBAR1, PXR, VDR, and LXR in the colon. *n* = 6 per group. Statistical differences (a, c, and d) were calculated by Mann-Whitney *U*-test with FDR correction, and statistical differences (f) calculated by one-way ANOVA with Tukey’s *post hoc* test. **P < 0.01, *P < 0.05, and ^#^*P* < 0.10.

To validate whether the SFMT-induced colonic inflammation and cell variation were mediated by specific human-associated SBA-producing microbes, gut microbiota composition was again analyzed in recipient mice receiving SFMT and HFMT. The compositional evaluation showed an apparent difference between the two groups at the phylum, genus, and feature layers (Supplementary Figure S8). Similarly, although no change in alpha diversities was seen (Figure 7a), PCoA showed a striking discrepancy in overall community structures between the two groups (Figure 7b). Furthermore, a cluster of bacterial features involved in the production of SBAs, mainly including F64 (*Eubacterium-coprostanoligenes-group*), F91 (*Bacteroides*), F110 (*Lachnospiraceae-NK4A136-group*), F112 (*Alistipes*), F173 (*Lachnospiraceae-NK4A136-group*), F236 (*Bacteroides*), and F279 (*Lachnospiraceae-NK4A136-group*), were almost eliminated by SFMT (Figure 7c). These features, it should be noted, are also absent in the intestine of SUCs from our clinical cohorts.

Additionally, a substantial reduction of the two dominant SBAs (DCA and 12-KLCA) was observed in the intestine of mice with SFMT (Figure 7d), consistent with our clinical results. Moreover, Spearman’s correlation analysis testified that 12-KLCA exhibited a positive relationship with ILC3s, with no significant correlation between 12-KLCA and Th17 cells (Figure 7e). The overall expressions of several critical BARs in the colon, including FXR, GPBAR1, PXR, VDR, and LXR, were subsequently detected. Among these receptors, the expressions of PXR and VDR were strongly down-regulated by SFMT relative to HFMT (Figure 7f). Overall, these observations support a potential association between the microbiota-derived bile acid profiles (especially 12-KLCA) and UC exacerbation episodes that might mediate the differentiation of ILC3s via regulating the expression of PXR and/or VDR.

### 12-KLCA but not DCA contribute to an anti‑inflammatory phenotype in DSS‑induced colitis

**Figure 8.**
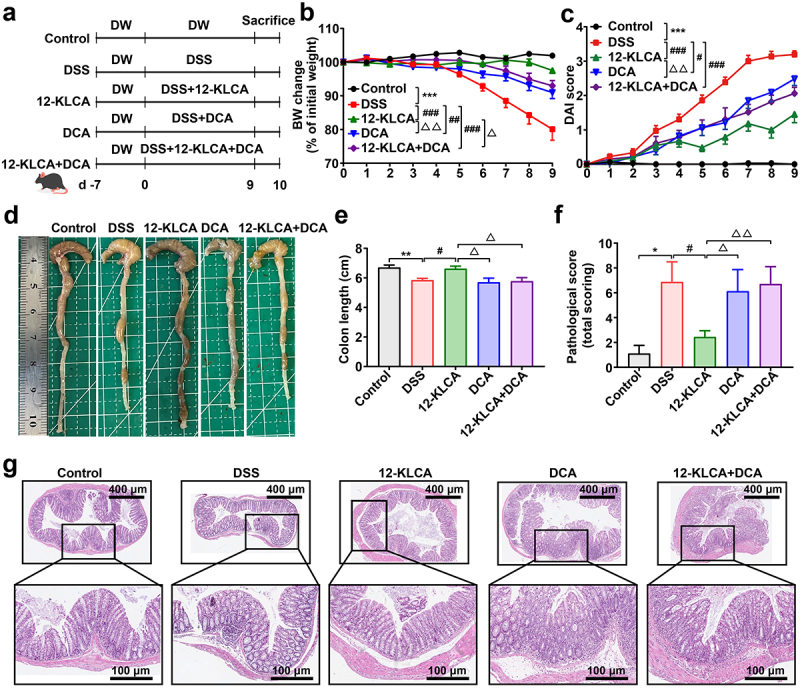
Oral administration of 12-KLCA ameliorated DSS-induced acute colitis in mice. (a) the protocol of animal treatments and sample collection. (b) body weight change of mice throughout the entire trial; *n* = 11 per group. (c) DAI score; *n* = 11 per group. (d) Representative images of the colon. (e) colonic length; *n* = 4 ~ 5 per group. (f) a summarized histopathological score of the colon. (g) H&E staining of the colon; *n* = 7 ~ 9 per group from two batches of mice. Statistical differences (b, c, e, and f) were calculated by one-way ANOVA with Tukey’s *post hoc* test. ***P < 0.001, **P < 0.01, and *P < 0.05, compared with the control; ^###^*P* < 0.001, ^##^*P* < 0.01, and ^#^*P* < 0.05, compared with the DSS group; ^△^^△^*P* < 0. 01 and ^△^*P* < 0.05, compared with the 12-KLCA group.

Given that 12-KLCA and DCA were the two SBAs with the most pronounced changes caused by the gut microbiota from SUCs, we finally attempted to explore whether they could directly prevent colonic inflammation. A DSS-induced colitis mouse model was employed and received the oral administration of 12-KLCA, DCA, and a combination of both at the beginning of 9 days of DSS treatment (Figure 8a). The data indicated that colitis mice with 12-KLCA, DCA, and 12-KLCA+DCA treatments exhibited significant body weight recovery (Figure 8b) and lower disease activity index (DAI) (Figure 8c) than the model control. In particular, 12-KLCA resulted in the highest weight recovery and the lowest DAI index compared to DCA and/or 12-KLCA+DCA. Furthermore, 12-KLCA induced a longer colonic length in colitis mice than in the model, DCA, and 12-KLCA+DCA groups (Figure 8d,e). In agreement with these outcomes, 12-KLCA dramatically reduced pathological scores by inhibiting expanded inflammatory cell infiltration and epithelial layer destruction in the colon of colitis mice (Figure 8f,g). However, DCA and 12-KLCA+DCA had no significant effect on the colonic length and pathological manifestation of colitis mice (Figure 8d-g). These phenotypic consequences collectively suggested that the supplementation of 12-KLCA achieved the desired ability to prevent colitis from worsening.

### 12-KLCA but not DCA attenuates the secretion of IL-17A by ILC3s via up-regulating the intracellular expression of VDR in DSS‑induced colitis mice

**Figure 9.**
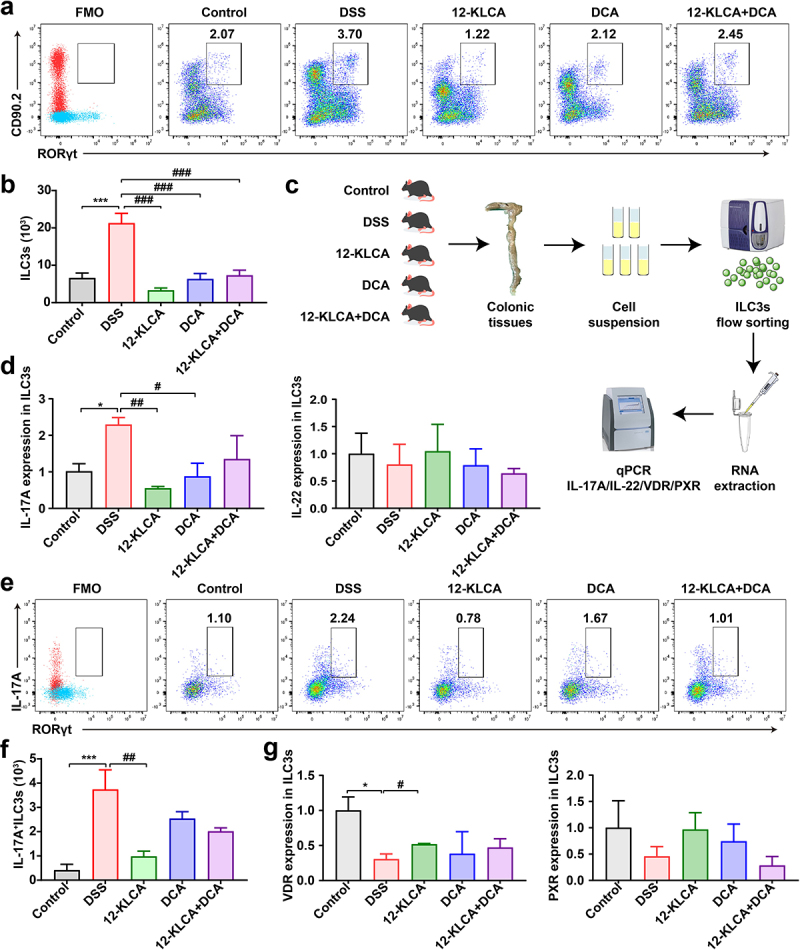
Oral administration of 12-KLCA inhibited the secretion of ILC3-derived IL-17A by increasing the VDR expression. (a) Representative scatter plots for identifying ILC3s in the colon gated by CD45^+^Lin^−^CD90.2^+^RORγt^+^cells. (b) the absolute number of ILC3s. *n* = 4 per group. (c) the process of ILC3s sorting *in vitro*. Live CD45^+^Lin^−^KLRG1^−^ILC3s were sorted from the colon; each sample here was sorted from the pooled colonic cells of every two mice per group. (d) the mRNA expressions of IL-17A and IL-22 in sorted ILC3s; *n* = 3 per group. (e) Representative scatter plots for identifying IL-17A^+^ILC3s in the colon gated by CD45^+^Lin^−^CD90.2^+^RORγt^+^IL-17A^+^cells. (f) the absolute number of IL-17A^+^ILC3s; *n* = 4 per group. (g) the mRNA expressions of VDR and PXR in sorted ILC3s; *n* = 3 per group. Statistical differences (b, d, f, and g) were calculated by one-way ANOVA with Tukey’s *post hoc* test. ***P < 0.01 and *P < 0.05, compared with the control; ^###^*P* < 0.001, ^##^*P* < 0.01, and ^#^*P* < 0.05, compared with the DSS group.

Since the absence of 12-KLCA in SFMT-derived recipients showed a positive association with the decrease of ILC3s, we further explored the impact of 12-KLCA on ILC3s in DSS-induced colitis. To our surprise, the percentage of ILC3s was increased in the DSS group, while 12-KLCA, DCA, and 12-KLCA+DCA reversed this change with 12-KLCA inducing the lowest value (Figure 9a,b), suggesting that these metabolites play their inflammatory regulatory roles at least in part through the regulation of ILC3s.

On the other hand, ILC3s are known as the main producers of type 3 cytokines IL-17A and IL-22 to play a significant role in the development of colitis.^21^ Therefore, to definite the functional cell subsets where ILC3s mediate the anti-inflammatory effects of 12-KLCA, we performed the *in vitro* sorting for the colonic ILC3s from all mice (Figure 9c). The mRNA expressions of IL-17A and IL-22 were further quantified in sorted ILC3s. As shown in Figure 9d, IL-17A mRNA was preferentially reduced by approximately 75% after 12-KLCA and DCA treatment, while IL-22 mRNA showed no difference between any two groups. Meanwhile, this finding was also confirmed by a drastic decrease of IL-17A^+^ILC3s in the colon of colitis mice receiving 12-KLCA compared with the DSS group (Figure 9e,f). No significant change in IL-17A^+^ILC3s was observed after DCA and 12-KLCA+DCA treatments. Finally, we tested the mRNA levels of PXR and VDR to determine whether 12-KLCA regulates ILC3s by facilitating the intracellular expression of specific BARs. Our data suggested that the VDR expression was down-regulated in ILC3s of DSS-treated mice, but this trend was still reversed by 12-KLCA treatment (Figure 9g). These results reveal that 12-KLCA plays a dominant role in modulating the secretion of IL-17A by ILC3s via increasing the expression of VDR, which is partially responsible for 12-KLCA in treating colitis.

## Discussion

Intestinal microbiota dysbiosis has been considered one of the most widely proposed etiological causes in UC,^28^ accompanied by the variations affecting metabolic profiles including the microbiota-derived BAs.^18^ Despite that, how the bile acid-associated changes across different stages of active UC remains unexplored. Here, we demonstrated that, in a subset of patients with UC, the deficiency of a cluster of SBA-producing microbes and SBAs contributes to the onset and even progression from mild, moderate, and severe UC. More importantly, we identified a promising potential of fecal BAs metabolites, in combination with bacterial indicators, for the noninvasive diagnosis of the progression of UC. Furthermore, we validated a consequential relationship among the gut microbiota, the BAs pool, and colonic inflammation using cross-species FMT. And then, we found that the oral administration of 12-KLCA, a derivate of LCA that is very little in SUCs and their recipient mice, could exert an anti-inflammatory effect on experimental colitis through the inhibition of IL-17A secreted by ILC3s. These findings reveal the microbiota-derived bile acid defects of severe UC and their contribution to host inflammation and ILC3s dysfunction, which ultimately can serve as a potential strategy for early interventions during the aggravation period of UC.

In our study, microbial alpha and beta diversities were significantly reduced in patients with UC compared with HCs, in agreement with previous research.^18,29^ Furthermore, we found that SUCs had the lowest alpha diversity and their bacterial community structure significantly differed from that of MiUCs and MoUCs. Unlike mild-to-moderate UC, severe UC is characterized by spontaneous bleeding and ulceration of the mucosa as well as high susceptibility to steroid-dependent opportunistic infection. It is clinically regarded as a medical emergency that requires hospitalization with medical therapy, such as intravenous corticosteroids, or even surgery. This may explain the progressive shifts of the overall diversity indices in response to increasing disease severity. Similarly, a more extensive cohort study of 428 pediatric UC patients also observed the declined microbial diversity associated with disease progression.^6^

At the taxonomic level, potentially pathogenic *Escherichia coli* within the phylum Proteobacteria were predominantly enriched in SUCs. Similar observations have been reported in past studies that indicated a microbial composition with a higher abundance of Proteobacteria^7^ and *Escherichia* ^8^ in patients with more severe disease courses. As such, existing data have reported the association between the high prevalence of *E. coli* and IBD.^30^ Excessive adherent *E.coli*, dependent on *Shiga* toxin and H7 flagellin, interacts with intestinal epithelial cells to activate the proinflammatory response.^31^ Our research, together with previous cohort studies, supports a pivotal role of *E. coli* in the exacerbation of UC. The translocation of these pathogens across the intestinal epithelium could be a critical step linked to the inflammatory process. *Enterococcus-faecalis* and *Lactobacillus-salivarius* as well as the *bsh* gene, have the most negligible yield in SUCs than other subjects. Certain microbial species from *Lactobacillus* and *Escherichia coli* encode the *bsh* gene to induce the deconjugation of PBAs,^11,13^ which might explain the exceedingly abundant free PBAs mainly containing CA, CDCA, and UDCA in SUCs.

Conversely, a small cluster of specific bacterial features was strikingly absent in UC patients, particularly SUCs. These microbes mainly consist of the members within Clostridials, including *Lachnospiraceae-NK4A136-group*, *Ruminococcus-torques-group*, Ruminococcaceae, *Eubacterium-coprostanoligenes-group*, *Blautia*, and *Subdoligranulum*, as well as two species within *Bacteroides*, including *Bacteroides-coprocola* and *Bacteroides-uniformis*. Schirmer et al.^6^ and Kedia et al.^8^ have also found that bacteria classified as Clostridia progressively decrease with the disease progression. Most members of Clostridial and Bacteroidaceae are equipped with 7α/β-dehydroxylation (*baiJ*) enzymes and therefore result in the transformation of PBAs to SBAs, as well as other derivatives, in the colon.^11,14^ As well, the *baiJ* gene copy numbers were significantly reduced in the intestinal tract of SUCs. Altogether, our discoveries showed that the insufficiency in these *baiJ*-active bacterial indicators might cause a simultaneous disruption of the host BAs pool. It will be meaningful to explore the potential of *baiJ*-positive bacterial species for diagnosing and treating SUC.

Indeed, our untargeted metabolomics combined with pathway enrichment analysis indicated that the bile acid biosynthesis was altered in UC patients compared to HCs. Further targeted BAs profiling revealed that with the aggravation of UC, PBAs (such as CA and CDCA) accumulated excessively, while SBAs (such as DCA, LCA, and their derivates) were extensively deficient, with the most apparent changes in SUCs. These data supported the findings from another recent study about the difference in BA profiles between UC and healthy participants^18^. Moreover, our correlation analysis uncovered the significant positive relationships between these PBAs and PBA-associated bacteria as well as between SBAs and SBA-producing taxa. Therefore, UC-derived microbiota might have a poor ability to modify PBAs into SBAs. In keeping with our data, relative to controls with familial adenomatous polyposis, UC pouches exhibited the lower proportions of microbial *bai* operon that are required to convert PBAs to SBAs and SBA-producing Ruminococcaceae^17^. A follow-up *in vitro* trial validated that fecal microbes from these UC patients exhibited an impaired ability to produce LCA and DCA compared to their controls, further supporting our hypothesis. We subsequently found that the combination of 21 metabolites and 40 bacterial features, such as CA, CDCA, DCA, LCA, *Lachnospiraceae-NK4A136-group*, *Ruminococcus-torques-group*, *Eubacterium-coprostanoligenes-group*, *etc*, distinguished MiUCs and MoUCs from non-MiUCs and non-MoUCs with an AUC of 0.84 and 0.88, respectively. Interestingly, these signatures achieved a much higher discriminating power, with an AUC of 0.94, for classifying SUCs from non-SUCs, again demonstrating their great potential for the early noninvasive diagnosis of UC exacerbation.

We next transplanted the fecal microbial suspension from HCs, MiUCs, and SUCs to antibiotic-treated mice to study the effects of the deficiency of mixed SBA-producing microbes and SBAs on the progression of colonic inflammation. Notably, only SFMT successfully reproduced the inflammatory phenotypes, including greater body weight loss, increased pathological damage, and excessive pro-inflammatory cell infiltration in the colon of recipients. However, MiUCs failed to induce the above pathological defects. Similar to our clinical observations, SFMT also resulted in a significant lack of *Eubacterium-coprostanoligenes-group*, *Bacteroides*, *Lachnospiraceae-NK4A136-group*, and *Alistipes* in the intestine of recipients, accompanying an almost vanishment of DCA and 12-KLCA. These outcomes demonstrated the vital contributions of the microbiota-derived SBA deficiency to determining the inflammatory responses of the host.

Intestinal microorganisms have complicated communications with ILC3s that are critical for intestinal homeostasis, tissue repair, host defense, and even the regulation of adaptive immunity, through microbial metabolites.^20,21^ Previous publications have observed fewer ILC3s in the colonic mucosa of individuals with UC as compared to HCs using single-cell sequencing,^2,25^ which is consistent with our observation in mice colonized with SUC-derived microbiota. Beyond that, our association analysis revealed that ILC3s were positively associated with 12-KLCA in recipients receiving SFMT, implicating the possible significance of 12-KLCA in regulating ILC3s to alleviate colitis. Therefore, we selected 12-KLCA for subsequent investigations in this study.

12-KLCA is a microbiota-produced derivative of LCA. Here, we demonstrated for the first time that 12-KLCA administration strongly alleviated DSS-induced colonic inflammation, consistent with several recent investigations showing the resistance to colitis by other SBAs.^12,15,27,32,33^ Notably, our data showed DCA alone and in combination with 12-KLCA was less effective than 12-KLCA alone in restoring colonic length and reducing histopathological damage of DSS-treated mice. DCA has been demonstrated to facilitate high-fat diet-induced colonic inflammation by skewing M1 macrophage polarization and pro-inflammatory cytokines production.^34^ Some researchers have even reported the involvement of DCA in liver,^35,36^ gastric,^37,38^, and intestinal^39,40^ carcinogenesis. We therefore speculate that the side effects of DCA might counteract the therapeutic effect of 12-KLCA alone, ultimately leading to the relative ineffectiveness of the combination of the two SBAs. To our surprise, at the cellular level, 12-KLCA further inhibited the higher accumulation of colonic ILC3s in colitis mice which is contradictory to the observations in the FMT trial, indicating the pro-inflammatory tone of ILC3s in DSS-induced colitis. In various states of IBD, ILC3s have been regarded as a double-edged sword that can produce classical cytokines, mainly including IL-17A and IL-22, to mediate the pathological progress of the inflamed intestine.^20,21,24^ In this study, for the first time, we linked the role of BAs and ILC3s in colitis and found that the 12-KLCA-induced improvement of colitis was associated with the preferential suppression of ILC3s secreting IL-17A, but not IL-22.

Moreover, BAs exert immunomodulatory actions by binding to their receptors expressed on immune cells in the IBD pathogenesis.^11,41^ For example, our data further suggested the decreased colonic expressions of the receptor VDR, in the colon of mice receiving SFMT, consistent with previous clinical investigations.^18^ Intestinal ILC3s have been evidenced to express a high level of VDR.^42^ We subsequently discovered that the ILC3s of DSS-treated mice had a much lower expression of VDR but 12-KLCA treatment significantly reversed this change. A previous study has shown that the ILC3-targeted deletion of VDR could increase the host susceptibility to pathogenic infection.^42^ Our results uncover that VDR might serve as a critical mediator for the significant interplay between 12-KLCA and colonic ILC3s in UC. Nevertheless, it is worth noting that the involvement of other microbial metabolites as the ligands of BARs in intestinal homeostasis and inflammation has also been extensively studied. For instance, indole-3-propionic acid (IPA), produced by the metabolism of tryptophan by Clostridial commensals, acts as another ligand for PXR and can reduce intestinal inflammation in a PXR-dependent manner.^43–46^ In this study, we failed to observe the up-regulation of the PXR expression in response to 12-KLCA. However, less abundant IPA-producing Clostridial commensals in SUCs implicate the possible involvement of IPA binding to PXR in anti-inflammatory response, which deserves further investigation.

One limitation of this study was the lack of absolutely germ-free models, such as gnotobiotic pigs or mice, to perform inter-species FMT to better exclude the effects of native microbiota of recipients. Complex bacterial isolates mediating the 12-KLCA synthesis from UC donors, as alternatives to FMT, can be used to colonize germ-free animals to fully validate the link between microbiota, 12-KLCA, and ILC3s during the UC progression. Another limitation is that the IL-17A^+^ILC3s-dependent pattern of 12-KLCA alleviating colitis requires in-depth validation by cell adoptive transfer or antibody neutralization. Future studies would also utilize the gene conditional knockout to clarify the targeting role of VDR in 12-KLCA regulating IL-17A^+^ILC3 and even decipher their transcriptional regulatory profiles through single-cell spatial transcriptomics.

**Figure 10.**
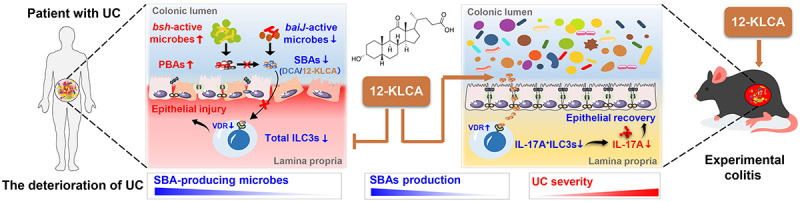
Integrative diagram showing the main results obtained from the present work.

In conclusion (Figure 10), our clinical cohort demonstrates that the gut microbiota structure of SUCs differs significantly from that of MiUCs and MoUCs, implicating the dominant role of microbiota in the development of mild, moderate, and severe UC. The deficiency of SBA-producing bacterial features encoding *baiJ* genes, such as *Eubacterium-coprostanoligenes-group*, *Bacteroides*, *Lachnospiraceae-NK4A136-group*, and *Alistipes*, together with the lack of microbiota-derived bile acid signatures, mainly including 12-KLCA, contributes to the exacerbation of UC. Moreover, we show for the first time that a novel microbiota-derived metabolite, 12-KLCA, exhibits a powerful anti-inflammatory effect in DSS-induced colitis by increasing the VDR expression and decreasing the IL-17A secretion in colonic ILC3s. Further follow-up studies will focus on elucidating which bacterial species mediate the production of 12-KLCA and how 12-KLCA interacts with IL-17A^+^ILC3s via the specific receptor to prevent the onset and exacerbation of UC. These findings, based on three cohorts of mild, moderate, and severe UC, further complement existing clinical studies that lack the indicators of various degrees of UC severity and emphasize mixed UC. To our knowledge, this is the first investigation that illustrates a direct association between microbiome, SBAs, and ILC3s involved in the deterioration of UC. Microbial-mediated bile acid metabolism warrants more consideration as a novel noninvasive signature test for the monitoring, prevention, and therapy of the acute deterioration of UC. Moreover, the selective modulation of the biosynthesis or distribution of SBAs such as 12-KLCA may be a corresponding potential therapeutic strategy in the future.

## Materials and methods

### Subject recruitment and sample collection

A total of 150 human subjects (29 MiUCs, 79 MoUCs, 25 SUCs, and 17 HCs) were recruited from 11 medical institutes (Supplementary Methods) according to the inclusion and exclusion criteria described in Table 1. The clinical and pathologic characteristics of all subjects are reported in Table 2. The disease activity of UC was assessed using a modified total Mayo score of 0 ~ 12, which mainly included a measure of defecation frequency, rectal bleeding, colonoscopic inflammation, and physician assessment.^3^ A Mayo score was 3 ~ 5 for MiUCs, 6 ~ 10 for MoUCs, and 11 ~ 12 for SUCs. Fecal specimens were collected from all participants, snap-frozen immediately, and stored at −80 °C until DNA and metabolite extraction. All participants provided written informed consent. Clinical trial registries can be retrieved at http://www.chictr.org.cn/index.aspx (Registration No. ChiCTR1900024591, ChiCTR1900023159, ChiCTR1900023158, and ChiCTR2000041225). All clinical protocols were approved by the Ethics Committees of Peking University First Hospital, Dong Fang Hospital, and Jiangsu Hospital of Chinese Medicine (JDF-IRB-2019030201).

**Table 1.** Inclusion and exclusion criteria.

Healthy controls (HCs) inclusion criteria
• Enrolled HCs were subjects of both sexes, aged 18 to 70 years, Chinese nationality, no relatives with UC, and who did not present evidence of illness based on the anamnestic data collected
Patients with different disease activities (MiUCs, MoUCs, and SUCs) inclusion criteria
• Patients with active disease were subjects of both sexes, and 18 to 70 years old
• Diagnosis was in line with the active period of UC
• Patients with a total Mayo score of 3 ~ 5 were defined as MiUCs, 6 ~ 10 as MoUCs, and 11 ~ 12 as SUCs
Patients with UC exclusion criteria
• Pregnancy, lactation, or recent family planning
• Allergic constitution
• Complicated with serious primary diseases such as cardiovascular, cerebrovascular, liver, kidney, and hematopoietic system
• Other serious intestinal diseases
• Patients with mental disorders and mental disorders
• Patients receiving other treatments for UC except for mesalazine

**Table 2.** Clinical characteristics of clinical subjects.

Items	Patients with different degrees of UC			HCs (*n* = 17)
	MiUCs (*n* = 29)	MoUCs (*n* = 79)	SUCs (*n* = 25)	
Age, years	44 (26 ~ 63)	39 (18 ~ 66)	46 (24 ~ 68)	37 (22 ~ 68)
Gender (M/F)	15/14	58/21	18/7	6/11
Disease duration	61.3 (4–240)	44.1 (1–276)	64.7 (1–240)	/
Disease extent, months				
• Proctitis	19 (65.5%)	31 (39.2%)	0 (0.0%)	/
• Left-sided colitis	3 (10.3%)	29 (36.7%)	5 (20%)	/
• Pancolitis	7 (24.1%)	19 (24.1%)	20 (80%)	/
Total Mayo score	3.9 (0.1)	7.8 (0.1)	11.6 (0.1)	/
IBDQ score	179.3 (3.9)	127.7 (4.2)	127.9 (8.9)	206.9 (3.3)
• Bowel symptoms	55.4 (1.2)	39.3 (1.2)	37.6 (3.3)	66.9 (1.0)
• Systemic symptoms	26.7 (0.8)	19.7 (0.7)	19.3 (1.5)	29.3 (1.0)
• Affective function	66.4 (1.9)	47.2 (1.7)	54.7 (3.8)	76.2 (2.2)
• Social function	30.7 (0.6)	21.6 (0.8)	16.3 (1.7)	34.6 (0.2)
Clinical features				
• Rectal bleeding	26 (89.7%)	77 (97.5%)	25 (100%)	/
• Diarrhea	18 (62.1%)	79 (100.0%)	25 (100%)	/
• Urgency	11 (37.9%)	65 (82.3%)	22 (88.0%)	/
• Tenesmus	14 (48.3%)	72 (91.1%)	22 (88.0%)	/
• Abdominal pain	17 (58.6%)	71 (89.9%)	24 (96.0%)	/
• Fever	0 (0.0%)	1 (1.3%)	13 (52.0%)	/
Endoscopic features				
• Loss of vascular texture	16 (55.2%)	45 (57.0%)	24 (96.0%)	/
• Erythema	4 (13.8%)	30 (38.0%)	23 (92.0%)	/
• Granularity	13 (44.8%)	19 (24.1%)	19 (76.0%)	/
• Friability	8 (27.6%)	49 (62.0%)	21 (84.0%)	/
• Erosions	21 (72.4%)	66 (83.5%)	23 (92.0%)	/
• Ulcerations	15 (51.7%)	62 (78.5%)	21 (84.0%)	/
• Spontaneous bleeding	2 (6.9%)	19 (24.1%)	11 (44.0%)	/
Pathological features				
• Distortion of crypt architecture	4 (13.8%)	20 (25.3%)	11 (44.0%)	/
• Crypt abscesses	3 (10.3%)	13 (16.5%)	22 (88.0%)	/
• Lamina propria cellular infiltrate (plasma cells, eosinophils, and lymphocytes)	24 (82.8%)	68 (86.1%)	24 (96.0%)	/
• Shortening of the crypts	5 (17.2%)	14 (17.7%)	11 (44.0%)	/
• Mucin depletion	2 (6.9%)	23 (29.1%)	3 (12.0%)	/
• Lymphoid aggregates	7 (24.1%)	41 (51.9%)	23 (92.0%)	/
• Erosion or ulceration	23 (79.3%)	39 (49.4%)	21 (84.0%)	/

### Human-associated FMT treatment in an antibiotic-treated mouse model

We subsequently conducted human-associated FMT based on an antibiotic-treated mouse model to explore the pro-inflammatory mechanisms of microbiota from patients with different degrees of UC. Three groups of donors were randomly selected from HCs, MiUCs, and SUCs to ensure that they had essentially the same characteristics as their respective clinical cohorts. Firstly, the microbial suspension of human donors was prepared as previously described.^47^ Briefly, about 1,000 mg clinical stool sample was weighted and pooled from each of MiUCs, SUCs, and HCs donors (*n* = 15 ~ 20), homogenized, and diluted 10-fold in sterile phosphate buffer saline (0.1 M, pH 7.2) containing 15% glycerol (v/v) under strictly anaerobic conditions. After centrifugation at 500 × g for 5 min, the fecal supernatant was passed through 100 μm strainers, snap-frozen, and stored at −80 °C for FMT treatment.

Six-week-old male C57BL/6 mice of similar body weights were purchased from SPF Biotechnology Co., Ltd. (Beijing, China) and maintained under specific pathogen-free (SPF) conditions on a 12 hr light-dark cycle of 25 °C with free access to irradiated chow and autoclaved water. To neutralize the effects of innate intestinal microbiota, mice were orally administrated with a broad-spectrum antibiotic cocktail containing vancomycin (100 mg/kg; V2002; Sigma-Aldrich), neomycin sulfate (200 mg/kg; 1758; INALCO), ampicillin (200 mg/kg; A9518; Sigma-Aldrich), and metronidazole (200 mg/kg; M1547; Sigma-Aldrich) once a day for 21 days after a one-week adaptation.^48^ Following the ABT, mice were randomly allocated into 3 groups based on similar body weights with 6 mice per cage in each group. Next, 200 μL of human microbial suspensions from HCs, MiUCs, and SUCs, were orally gavaged to these mice once a day for 19 days, respectively. After the FMT, mice were sacrificed, and feces were sampled from all recipients for 16S rRNA sequencing and BAs extraction. Colonic tissues were obtained for pathologic evaluation and flow cytometry. The animal study routine was approved by the Ethics Committee of the Beijing University of Chinese Medicine (BUCM-4-2021092705–3163).

### Oral supplementation of DCA and 12-KLCA in a DSS-induced colitis mouse model

Six-week-old male C57BL/6 mice were randomly allocated into four groups including control, DSS, DSS+12-KLCA, DSS+DCA, and DSS+12-KLCA+DCA with 5 ~ 6 mice per cage in each group. Except for the control, acute colitis was induced in the other three groups by the administration of 3% DSS (160110; MP Biomedicals) to drinking water *ad libitum* for 7 days followed by 1% DSS for 2 days. On days 0 to 10, mice in DSS+12-KLCA and DSS+DCA groups were orally administered with 2 mM of 12-KLCA (Purity > 95%; SMB00913; Sigma-Aldrich) and DCA (Purity > 99%; 30960; Sigma-Aldrich) each day, respectively, for consecutive 9 days. Meanwhile, mice in DSS+12-KLCA+DCA received a 2 mM mixture of 12-KLCA and DCA. Mice in the control and DSS groups were orally treated with equivalent sterile water. The severity of colitis was evaluated using the DAI score as described previously.^49^ In brief, each mouse was recorded daily for body weight loss, stool consistency, and fecal occult blood, and the DAI score was equal to the average of the above three indicators. In the end, mice were sacrificed, and the colonic length was measured. Colonic tissues were obtained for pathologic evaluation and flow cytometry. All experimental procedures were approved by the Ethics Committee of the Beijing University of Chinese Medicine (BUCM-2022120702–4088).

### DNA extraction, 16S rRNA gene sequencing, and data analysis

Total genomic DNA was extracted from the feces of all subjects and mice using the QIAamp® Fast DNA Stool Mini Kit (51604; Qiagen) following the manufacturer’s protocol. The V3-V4 hypervariable region of the bacterial 16S rRNA gene was amplified with universal primers 343F (TACGGRAGGCAGCAG) and 798 R (AGGGTATCTAATCCT). After purification and quantification, the PCR products were pooled into equal molar amounts and sequenced on an Illumina NovaSeq6000 platform to obtain paired-end reads of 250 bp.

Raw sequences were analyzed demultiplexed, adapter-trimmed, quality-filtered, denoised, assembled, and imported into the latest QIIME2 platform as previously described.^47^ Unique amplicon sequence variants (ASVs) or bacterial features were generated after the removal of chimeric sequences using the DADA2 plugin.^50^ To minimize the effects of sequencing depth, we randomly rarefied per sample to 21,111 sequences for downstream analysis. The SILVA reference database classifier (version 132) was used for the classification of ASVs with 99% similarity. Alpha and beta diversities were also calculated in QIIME2. PERMANOVA (1,000 Monte Carlo permutations) based on Bray-Curtis and Jaccard dissimilarities was performed to disclose the microbial differences between groups with the Adonis function in the “vegan” package of R software (version 3.3.1, https://www.r-project.org/).^51^ PCoA based on the above-mentioned distance metrics was carried out to visualize the overall difference in microbiota structures among groups. Differentially abundant features between groups were identified using LEfSe (LDA score > 2). Regression-based random forest models were employed to explore indicator features related to the disease severity using the default settings in the randomForest package of R.^52^ Microbial ecological networks among the specific microbes were estimated and visualized using the SPIEC-EASI algorithm and the igraph package in R, respectively. Clusters were generated based on the betweenness centrality calculated with the Girvan-Newman algorithm.^53^ Spearman correlation coefficients between microbes and metabolites were inferred using the “psych” package in R. Bar plots and heat maps were also visualized using the “ggplot2” and “pheatmap” packages in R, respectively. Potential bacterial and metabolite biomarkers were verified by random forest models with 10-fold cross-validation and the receiver operating characteristic (ROC) curve was then generated to illustrate the performances of classification models, using the “caret” and the “pROC” packages in R, respectively.

### Fecal metabolite extraction, untargeted metabolomics detection, and data processing

We next performed fecal untargeted metabolomics to investigate disease-related metabolites in the intestine of patients with UC, based on a previous publication with slight adjustments.^54^ About 100 mg of each fecal sample (29 MiUCs, 24 MoUCs, 25 SUCs, and 17 HCs) was mixed with 400 μL pre-cooling methanol and acetonitrile (1:1, v/v) containing L-2-chlorophenylalanine as an internal standard (0.3 mg/mL). After centrifugation, the supernatant was transferred and dried by a vacuum concentrator (Eppendorf, Germany). The dried extract was redissolved in 200 μL methanol:H_2_O (4:1, v/v, 4 °C) and centrifuged, and the final supernatant was passed through a 0.22 μm sterile membrane and detected using the ultra-performance liquid chromatography coupled to electrospray ionization tandem mass spectrometry (UPLC-ESI-MS/MS, Waters, U.S.A) under the standard procedures.

Original data were processed by the software Progenesis QI V2.3 for baseline filtering, peak identification, integral, retention time correction, peak alignment, and normalization. Primary parameters consisted of 5 ppm precursor tolerance, 10 ppm product tolerance, and 5% production threshold. Compound extraction was achieved based on the precise mass-to-charge ratio (M/Z), secondary fragments, and isotopic distribution using the Human Metabolome Database (HMDB) and Metlin to do qualitative analysis. OPLS-DA was performed using the MetaboAnalyst 5.0 cloud platform with the R package ropls inserted to distinguish the metabolites that differed between groups.^55,56^ Variable importance of projection (VIP) scores gained from the OPLS-DA matrix were selected to sort the overall contribution of each variable to group discrimination. The two-tailed Student’s *t*-test was further applied to identify differentially abundant metabolites with VIP > 1.0 and *P* values below 0.05.

### BA extraction and targeted quantification

Fecal BAs of human subjects (*n* = 20) and recipient mice (*n* = 6) were profiled as previously described.^16^ Briefly, one weighed fecal pellet was resuspended, sonicated, and homogenized in cold extraction solutions containing methanol and acetonitrile. After centrifugation, the supernatant was filtered through a 0.22 μm sterile membrane and analyzed using the UPLC-ESI-MS/MS system (Waters, U.S.A) under standard procedures. D4-cholic acid served as an internal standard. Forty corresponding synthetic standards (Supplementary Methods) were used to generate calibration curves for the concentrations of different BAs per sample.

### Total bacterial and microbial metabolic gene quantification by real-time PCR assay

The total bacteria, and the microbial metabolic genes involved in the conversion of PBAs and SBAs, mainly including *baiJ* and *bsh*, were quantified using QuantStudio6 Flex system (Life Technologies, U.S.A) with QuantiNova SYBR Green PCR Kit (208052; Qiagen). Fecal genomic DNA was extracted as mentioned above. PCR amplification was performed using bacterial group-specific primers as listed in Supplementary Table S2. The reaction mixture (25 μL) consisted of 1.5 μL forward and 1.5 μL reverse primers, 12.5 μL QuantiNova SYBR Green, 1 μL template DNA, and 8.5 μL ddH_2_O. The reaction procedure included one initial denaturation at 95 °C for 10 min, 40 cycles of denaturation at 95 °C for 10 s, 60 s at the appropriate annealing temperature (60 °C), and extension at 72 °C for 10 s. The copy numbers of total bacteria and microbial genes in the sample were calculated based on the corresponding standard curve. The target standard plasmids of total bacteria and microbial genes were constructed, and a series of continuous 10-fold dilutions (10^9^ to 10^1^ copies/μL) of the standard plasmid DNA was used to generate the standard curves. The logarithm of copy numbers was as the abscissa and the Ct values as the ordinate. The copy numbers were calculated using the equation below: (DNA concentration (μg/μL) × 6.0233 × 10^23^ copies/mol)/(DNA size (bp) × 660 × 10^6^. Each PCR reaction was set in triplicate.

### Histopathological examination

After the fixation with 10% formaldehyde for 24 h, colorectal tissues were paraffin-embedded, sectioned, and stained with hematoxylin and eosin (H&E) for histopathological analysis. Images of tissue morphology were obtained using a light microscope. The extent of tissue damage and inflammatory infiltration was scored as previously described.^57^

### Preparation of colonic single-cell suspensions, flow cytometry analysis, and ILC3 sorting

After the careful removal of adherent fat tissue and Peyer’s patches, colonic tissues were cut into small sections and washed twice with a 20 mL HBSS medium containing 5 mM EDTA and 1 mM DTT to remove epithelial cells. Subsequently, 2 mg/mL collagenase type III (LS004183; Worthington) and 50 μg/mL DNase I (10104159001; Roche) in RPMI-1640 medium were applied to digest colonic tissues. Digested colonic lamina propria cells were then filtered through 70 μm cell strainers to obtain final cell suspensions and isolated with a 40% isotonic Percoll density gradient separation after red blood cells were dissolved.

Single-cell suspensions were preincubated with purified anti-mouse CD16/32 at 4 °C for 10 min to block the nonspecific binding to Fc receptors before staining. Then, extracellular staining was performed according to the standard flow cytometry protocol (0.5 µL antibody/100 µL buffer/5 million cells). For ILCs surface staining, cells were pre-stained with biotin-conjugated lineage markers (Ly-6 G/Gr1/TCRγδ/TCRβ/TER-119/CD5/CD11b/CD8a/CD45R(B220)/CD19/CD3ε/CD4/CD11c), and subsequently stained with fluorochrome-conjugated antibodies. All anti-mouse antibodies were purchased from Biolegend (U.S.A) unless otherwise stated. For surface staining, the following antibodies were used: APC-Cy7-CD3 (100222), FITC-CD4 (100405), PB-CD45 (103126), FITC-CD45 (103107), FITC-Streptavidin (405201), APC-Cy7-Strapavidin (405208), AF700-CD90.2(105320), and APC-CD90.2 (105312). For the detection of intracellular factors, the obtained single-cell suspensions were first stimulated in culture for 4 ~ 6 h *in vitro* using Cell Stimulation Cocktail (500×) (00-4975-93, eBioscience) following the manufacturer’s instructions. Subsequently, cells were stained with intracellular antibodies: PE-RORγt (562607, BD Biosciences), PE-Cy7-T-bet (644824), APC-GATA3 (653806), BV421-RORγt (562894; BD Biosciences), and PE-IL-17A (506903). Single-stained positive tubes were set up to adjust the fluorescence compensation. Fluorescence minus one (FMO) was used to determine the gates. Stained cells were acquired on a Cytoflex flow cytometer (Beckman Coulter, U.S.A) and analyzed with FlowJo V10 software. The gating strategies for flow cytometry analysis are provided in Supplementary figure S9.

To sort live ILC3s, colonic cells were harvested and pooled from each two mice at each group per sorting session with a total of three sessions per group and sorted to > 95% purity. For sorting experiments, single-cell suspensions were stained with purified-CD16/32 (101302), biotin-conjugated lineage markers (Ly-6 G/Gr1/NK-1.1/TCRγδ/TCRβ/TER-119/CD5/CD11b/CD8a/CD45R(B220)/CD19/CD3ε/CD4/CD11c), FITC-CD45 (103107), APC-Cy7-Strapavidin (405208), APC-CD90.2 (105312), and PE-KLRG1(138408). Live CD45^+^Lin^−^KLRG1^−^ILC3s from mice were sorted to a purity > 99% by FACS (BD FACSMelody, U.S.A), and then harvested for immediate RNA extraction.

### RNA extraction from colonic tissues and ILC3s and real-time PCR assay

Total RNA from colonic tissues or ILC3s was extracted using PureLink RNA Mini Kit (12183016; Invitrogen). After the quantification and reverse transcription, cDNA transcripts were detected by qPCR with the specific primers of targeted cytokines (*IL-17A* and *IL-22*) and BARs (*FXR*, *GPBAR1*, *PXR*, *VDR*, and *LXR*). *GAPDH* was used as a reference gene. The relative mRNA expressions of targeted genes were calculated by the 2^−ΔΔCt^ method. Paired primers used in this research were listed in Supplementary Table S2.

### Statistical analysis

Statistical analysis was performed using SPSS for Windows (version 22.0, SPSS Inc., U.S.A). Parametric data were analyzed using an unpaired Student’s *t*-test or one-way analysis of variance (ANOVA) with Tukey’s *post hoc* test. Outliers were marked using the Descriptive Statistics module and the generated boxplots in SPSS 22.0. Non-parametric data were analyzed using the Mann-Whitney *U*-tests or Kruskal-Wallis test. *P* values for multiple comparisons were adjusted with FDR.^58^ Corrected *P* values less than 0·05 were considered significant. Data were presented as median or mean ± standard error of the mean (mean ± SEM).

## Supplementary Material

Revised supplementary tables and figures_20231122.docx

Supplementary methods_20231122.docx

## Data Availability

The raw untargeted and targeted metabolomics data supporting our conclusions have been deposited in the Metabolights database under project IDs: MTBLS7819 and MTBLS7825. The 16S rRNA amplicon sequencing data have also been deposited in the NCBI Sequence Read Archive (SRA) repository under BioProject PRJNA851926. These raw data will be available on July 31, 2024, or upon publication.

## List of abbreviations

ABT: antibiotic treatment
ANOVA: analysis of variance
AUC: area under the curve
BAs: bile acids
BA receptors: BARs
bsh: bile salt hydrolase
CA: cholic acids
CDCA: chenodeoxycholic acids
DCA: deoxycholic acids
EALCA: epiallolithocholic acids
FDR: false discovery rate
FMO: fluorescence minus one
FMT: fecal microbiota transplantation
FXR: farnesoid X receptor
GCA: glycocholic acids
GPBAR1: G protein-coupled bile acid receptor 1
HCs: healthy controls
H&E: hematoxylin and eosin
HFMT: HC-associated FMT
HDCA: hyodeoxycholic acids
IBD: inflammatory bowel disease
ILC3s: group 3 innate lymphoid cells
IPA: Indole-3-propionic acid
Iso-LCA: isolithocholic acids
LCA: lithocholic acids
LEfSe: linear discriminant analysis (LDA) effect size
LXR: liver X receptor
MiFMT: mild UC-associated FMT
MiUCs: patients with mild UC
MoUCs: patients with moderate UC
OPLS-DA: orthogonal partial least squares discriminant analysis
PBAs: primary BAs
PCoA: principal coordinates analysis
PERMANOVA: permutational multivariate analysis of variance
PXR: pregnane X receptor
SBAs: secondary BAs
SCFAs: short-chain fatty acids
SEM: standard error of the mean
SFMT: severe UC-associated FMT
SPF: specific pathogen-free
SUCs: patients with severe UC
TCA: taurocholic acids
UC: ulcerative colitis
UDCA: ursodeoxycholic acids
VDR: vitamin D receptor
3β-CA: 3β-cholic acids
12-KLCA: 12-ketolithocholic acids
