# Supplementary material for: Gut microbiota-derived 12-ketolithocholic acid suppresses the IL-17A secretion from colonic group 3 innate lymphoid cells to prevent the acute exacerbation of ulcerative colitis
Source: Gut Microbes. 2023 Dec 8;15(2):2290315. doi: 10.1080/19490976.2023.2290315 (PMC10730201; doi:10.1080/19490976.2023.2290315)
Supplement: Revised supplementary tables and figures_20231122.docx [file KGMI_A_2290315_SM3541.docx]

## Supplementary Tables

| \| Supplementary Table 1. Permutational multivariate analysis of variance (PERMANOVA, with 1,000 Monte Carlo permutations) based on Bray-Curtis and Jaccard dissimilarities \| \| \| \| \| \| \| --- \| --- \| --- \| --- \| --- \| --- \| \| Comparisons \| Unweighted Unifrac distances \| \|  \| Jaccard distances \| \| \| *R*^2^ value \| *P* value \|  \| *R*^2^ value \| *P* value \| \| HCs *vs*. MiUCs \| 0.161 \| 0.002 \|  \| 0.274 \| 0.001 \| \| HCs *vs*. MoUCs \| 0.084 \| 0.002 \|  \| 0.123 \| 0.001 \| \| HCs *vs*. SUCs \| 0.190 \| 0.001 \|  \| 0.291 \| 0.001 \| \| MiUCs *vs*. MoUCs \| 0.005 \| 0.505 \|  \| 0.018 \| 0.110 \| \| MiUCs *vs*. SUCs \| 0.055 \| 0.058 \|  \| 0.060 \| 0.014 \| \| MoUCs *vs*. SUCs \| 0.027 \| 0.075 \|  \| 0.055 \| 0.001 \|     Supplementary Table 2. Specific primer pairs used in this study | | | | |
| --- | --- | --- | --- | --- | --- | --- | --- | --- | --- | --- | --- | --- | --- | --- | --- | --- | --- | --- | --- | --- | --- | --- | --- | --- | --- | --- | --- | --- | --- | --- | --- | --- | --- | --- | --- | --- | --- | --- | --- | --- | --- | --- | --- | --- | --- | --- | --- | --- | --- | --- | --- | --- | --- | --- | --- | --- | --- |
| Items | Forward primer (5'-3') | Reverse primer (5'-3') | Annealing temperature | References |
| For bacteria |  |  |  |  |
| Total bacteria | ACTCCTACGGGAGGCAGCAG | ATTACCGCGGCTGCTGG | 60 ℃ | Li et al., 2020 |
| *bsh* | ATGGGCGGACTAGGATTACC | TGCCACTCTCTGTCTGCATC | 54 ℃ | Pi et al., 2023 |
| *baiJ* | TCAGGACGTGGAGGCGATCCA | TACRTGATACTGGTAGCTCCA | 60 ℃ | Pi et al., 2023 |
| For mouse |  |  |  |  |
| *IL-17A* | ATCCCTCAAAGCTCAGCGTGTC | GGGTCTTCATTGCGGTGGAGAG | 60 ℃ | Wu et al., 2021 |
| *IL-22* | ATGAGTTTTTCCCTTATGGGGAC | GCTGGAAGTTGGACACCTCAA | 60 ℃ | Song et al., 2020 |
| *FXR* | GAAAATCCAATTCAGATTAGTCTTCAC | CCGCGTGTTCTGTTAGCAT | 60 ℃ | Song et al., 2020 |
| *GPBAR1* | ATTCCCATGGGGGTTCTG | GAGCAGGTTGGCGATGAC | 60 ℃ | Song et al., 2020 |
| *PXR* | CAAGAGCGACGGGAAAGAGAT | CTTTGGCGAAGTTGATGACGC | 60 ℃ | Song et al., 2020 |
| *VDR* | CACCTGGCTGATCTTGTCAGT | CTGGTCATCAGAGGTGAGGTC | 60 ℃ | Song et al., 2020 |
| *LXR* | TGTGCGCTCAGCTCTTGT | TGGAGCCCTGGACATTACC | 60 ℃ | Song et al., 2020 |
| *GAPDH* | GCACCACCAACTGCTTAG | GGATGCAGGGATGATGTTC | 60 ℃ | Li et al., 2022 |

## Supplementary Figures


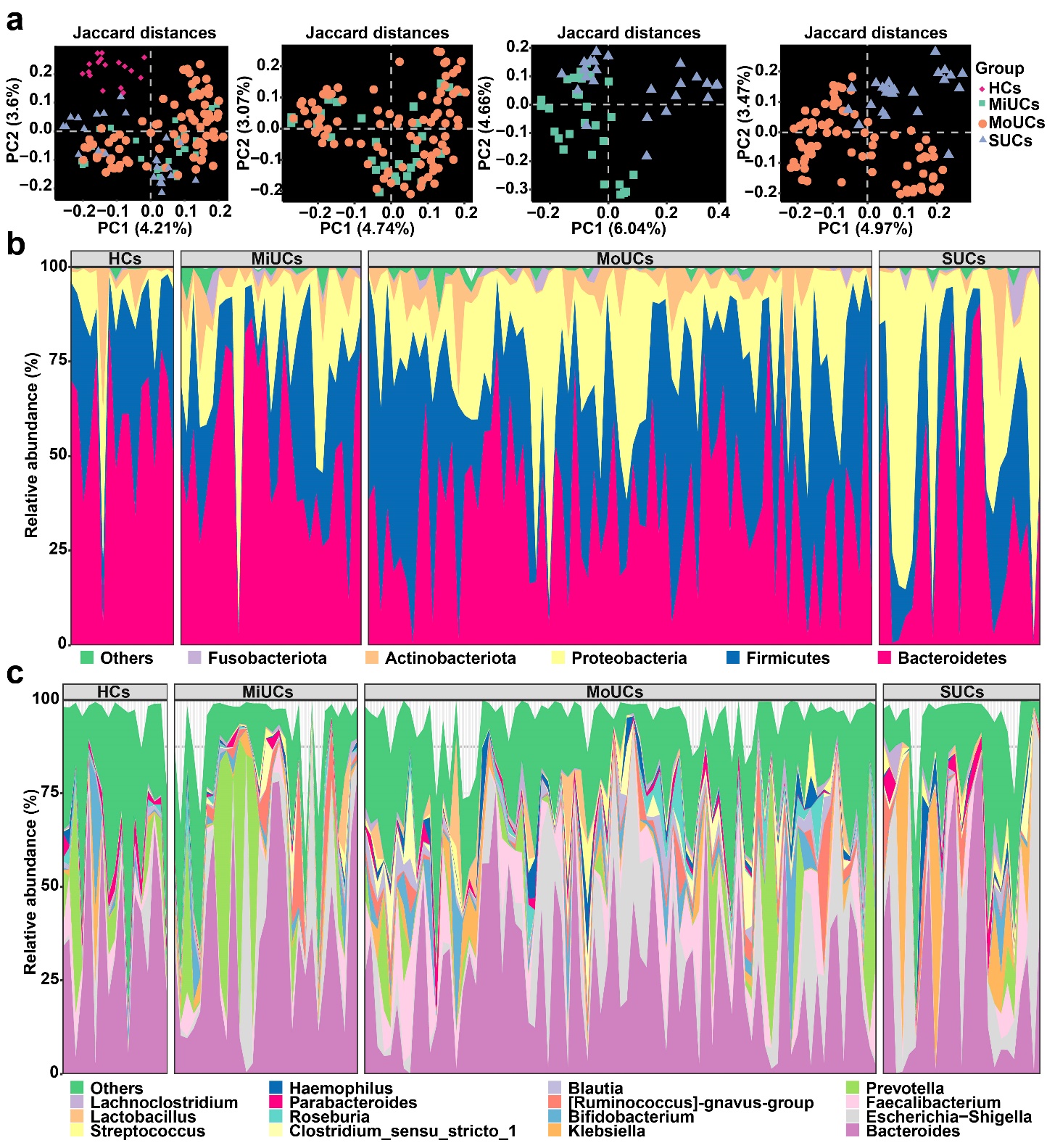


Supplementary Figure 1. Overall gut microbiota structure and composition of SUCs differed from MiUCs and MoUCs. (a) Beta-diversity of gut microbiota profiles illustrated with PCoA using Jaccard matrix. (b) Relative abundances of the top 5 phyla. (c) Relative abundances of the top 15 genera. HCs (n = 17), MiUCs (n = 29), MoUCs (n = 79), and SUCs (n = 25).


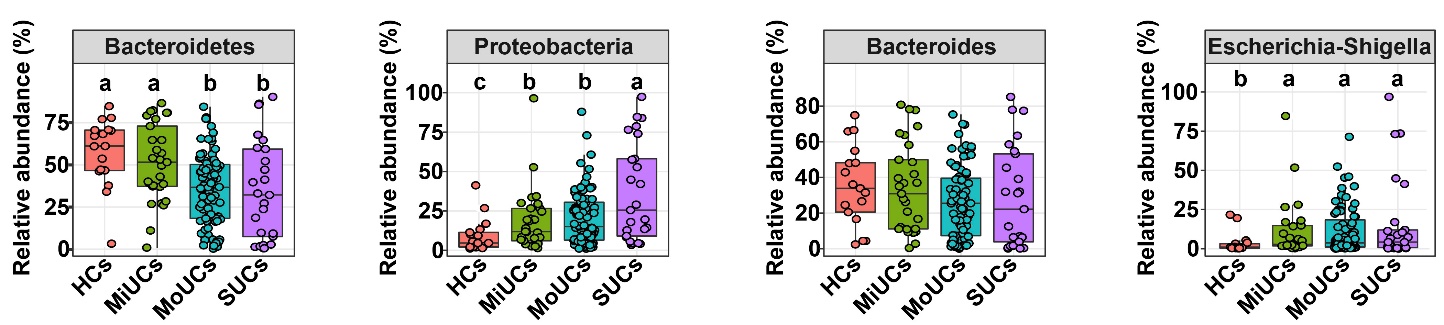


Supplementary Figure 2. Key phyla and genera that are significantly involved in the progression of UC. Statistical differences were calculated by Kruskal-Wallis test with false discovery rate (FDR) correction. ^a, b, c^ Different letters within each panel represent a significant difference (*P* < 0.05). HCs (n = 17), MiUCs (n = 29), MoUCs (n = 79), and SUCs (n = 25).


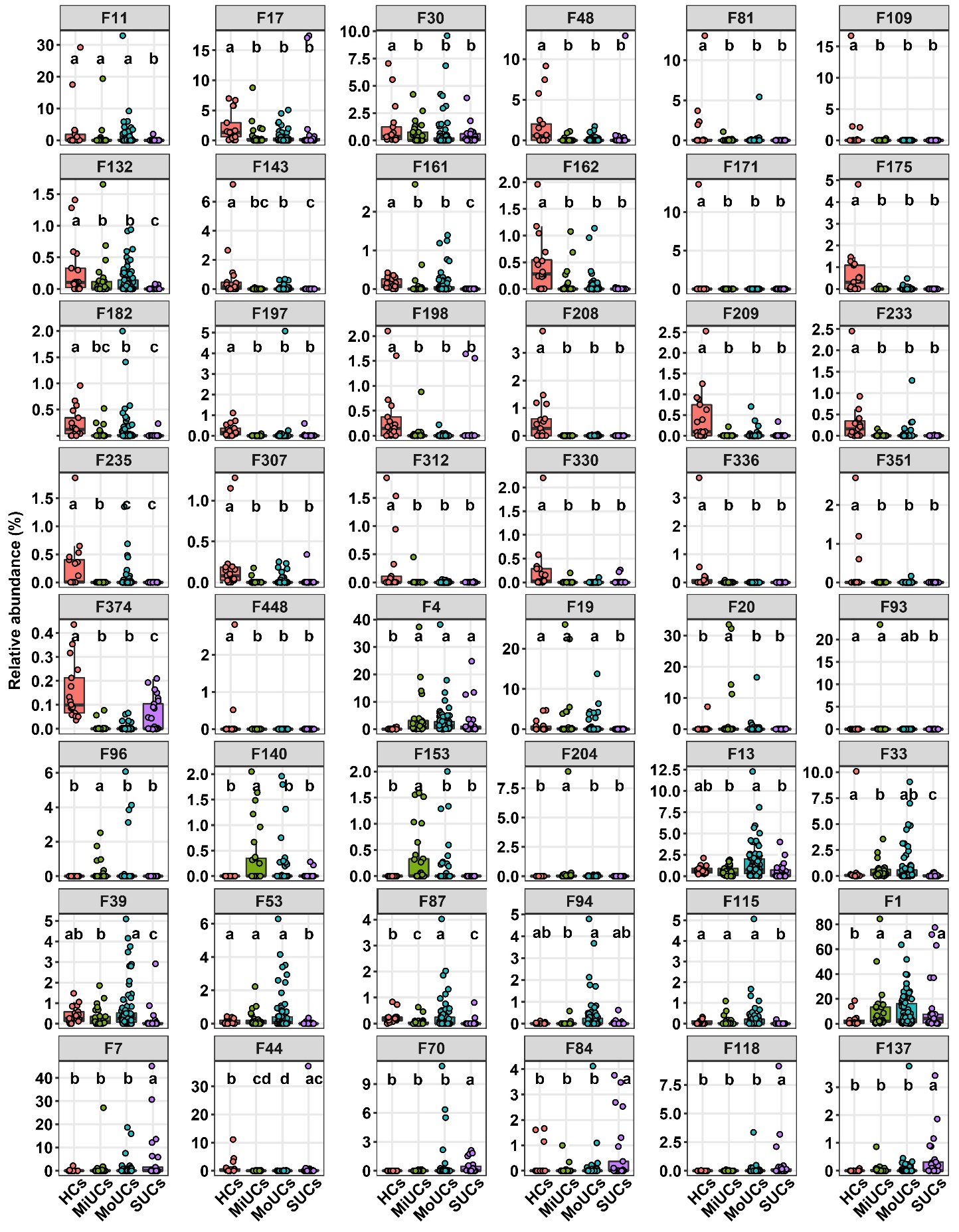


Supplementary Figure 3. Shared bacterial indicators of LEfSe and random forest involved in the deterioration of UC. Statistical differences were calculated by Kruskal-Wallis test with FDR correction. ^a, b, c, d^ Different letters within each panel represent a significant difference (*P* < 0.05). HCs (n = 17), MiUCs (n = 29), MoUCs (n = 79), and SUCs (n = 25).


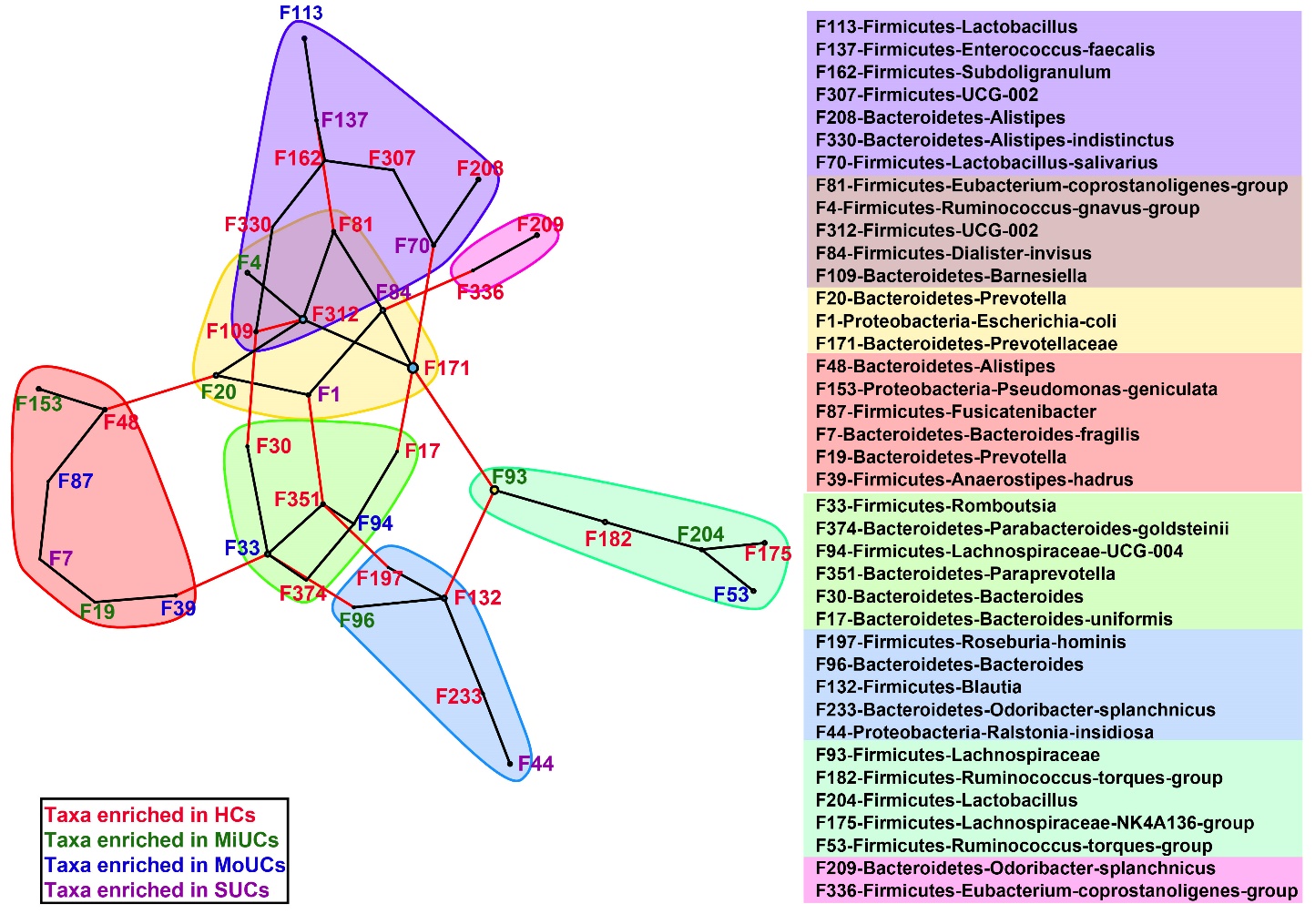


Supplementary Figure 4. Network analysis of the interactions between bacterial indicators involved in the progression of UC. SparCC was used to calculate the relationships between bacterial taxa. R package igraph was used to draw the network. HCs (n = 17), MiUCs (n = 29), MoUCs (n = 79), and SUCs (n = 25).


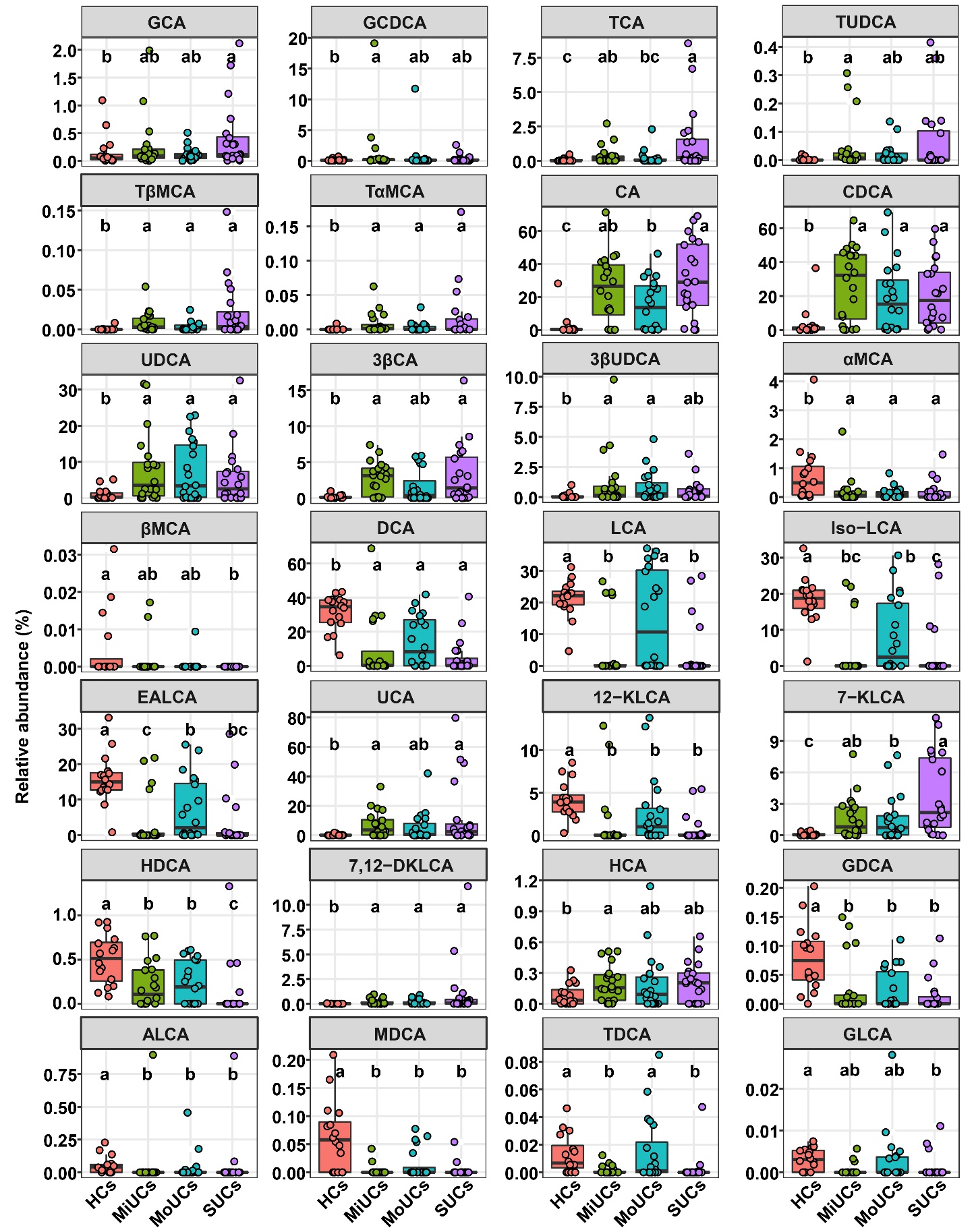


Supplementary Figure 5. Targeted metabolomics showing differentially abundant BAs among different groups. HCs (n = 16), MiUCs (n = 20), MoUCs (n = 20), and SUCs (n = 20). Statistical differences were calculated by Kruskal-Wallis test with FDR correction. ^a, b, c^ Different letters within each panel represent a significant difference (*P* < 0.05).


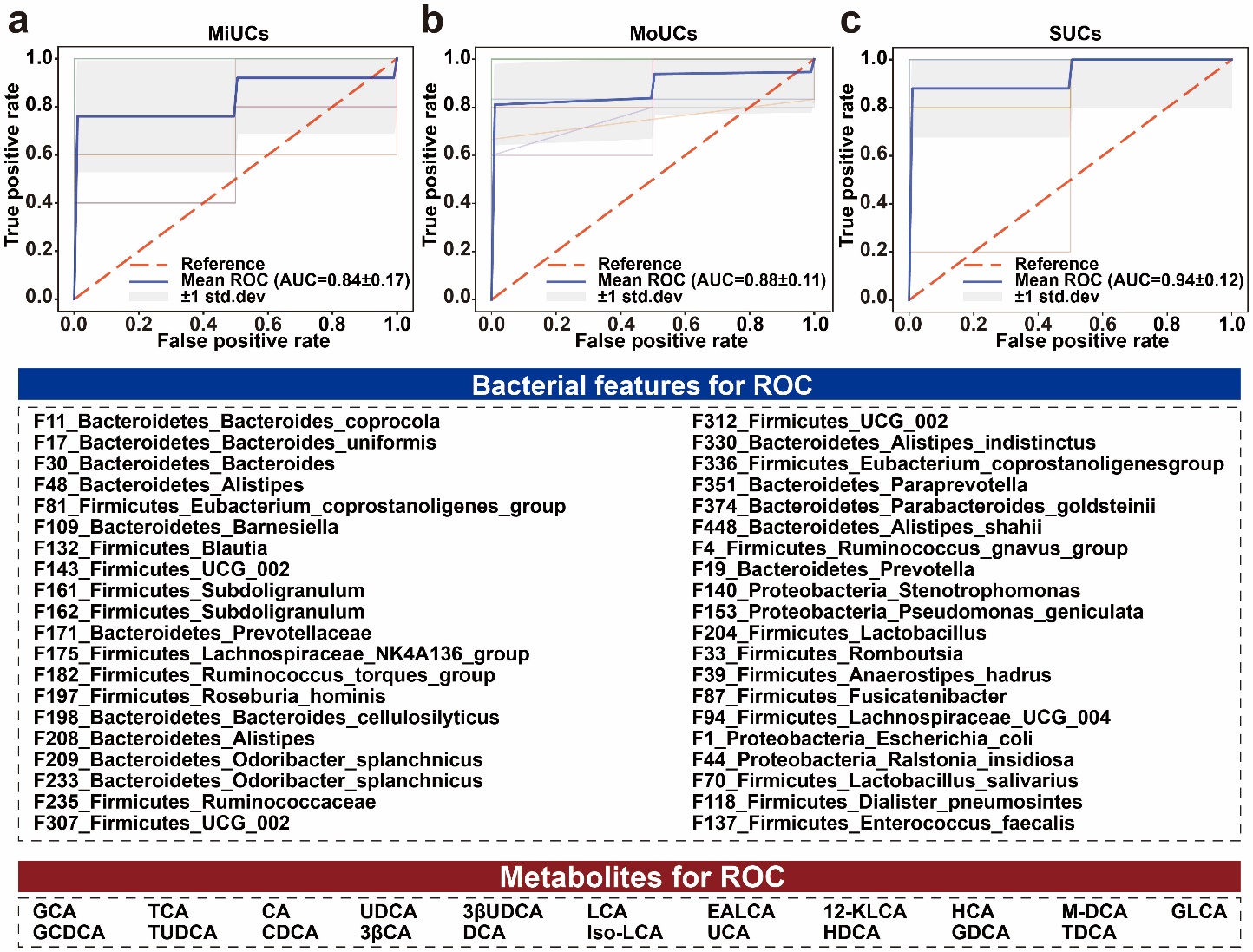


Supplementary Figure 6. Combination of bacterial and metabolite markers for pairwise discriminations of different degrees of UC. A total of 40 bacterial features and 21 metabolites were selected for the Receiver operating characteristic (ROC) analysis. (a) ROC curve for discriminating MiUCs from non-MiUCs. (b) ROC curve for discriminating MoUCs from non-MoUCs. (c) ROC curve for discriminating SUCs from non-SUCs.


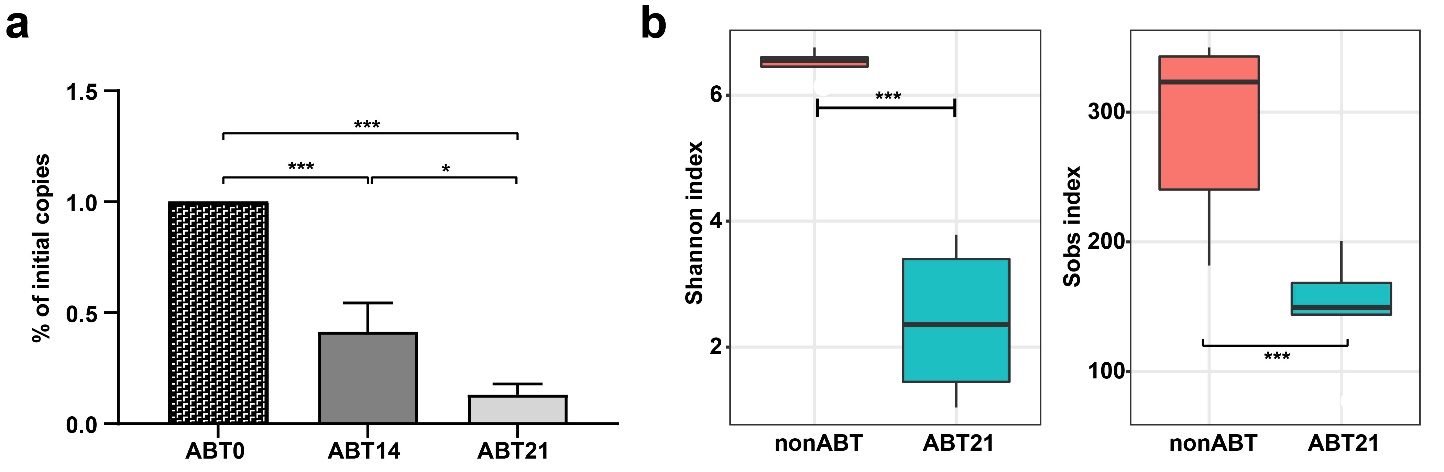


Supplementary Figure 7. An antibiotic cocktail achieved an ideal depletion of murine endogenous intestinal microbiota. (a) The copies of total bacteria of mice at 0, 14, and 21 days of ABT. Statistical differences were calculated by one-way ANOVA with Tukey’s *post hoc* test. (b) alpha diversity of mice at 0 and 21 days of ABT. Statistical differences were calculated by Mann-Whitney *U*-test with FDR correction. n = 13 at each time point with 3~4 mice randomly selected per group. ****P* < 0.001 and **P* < 0.05.


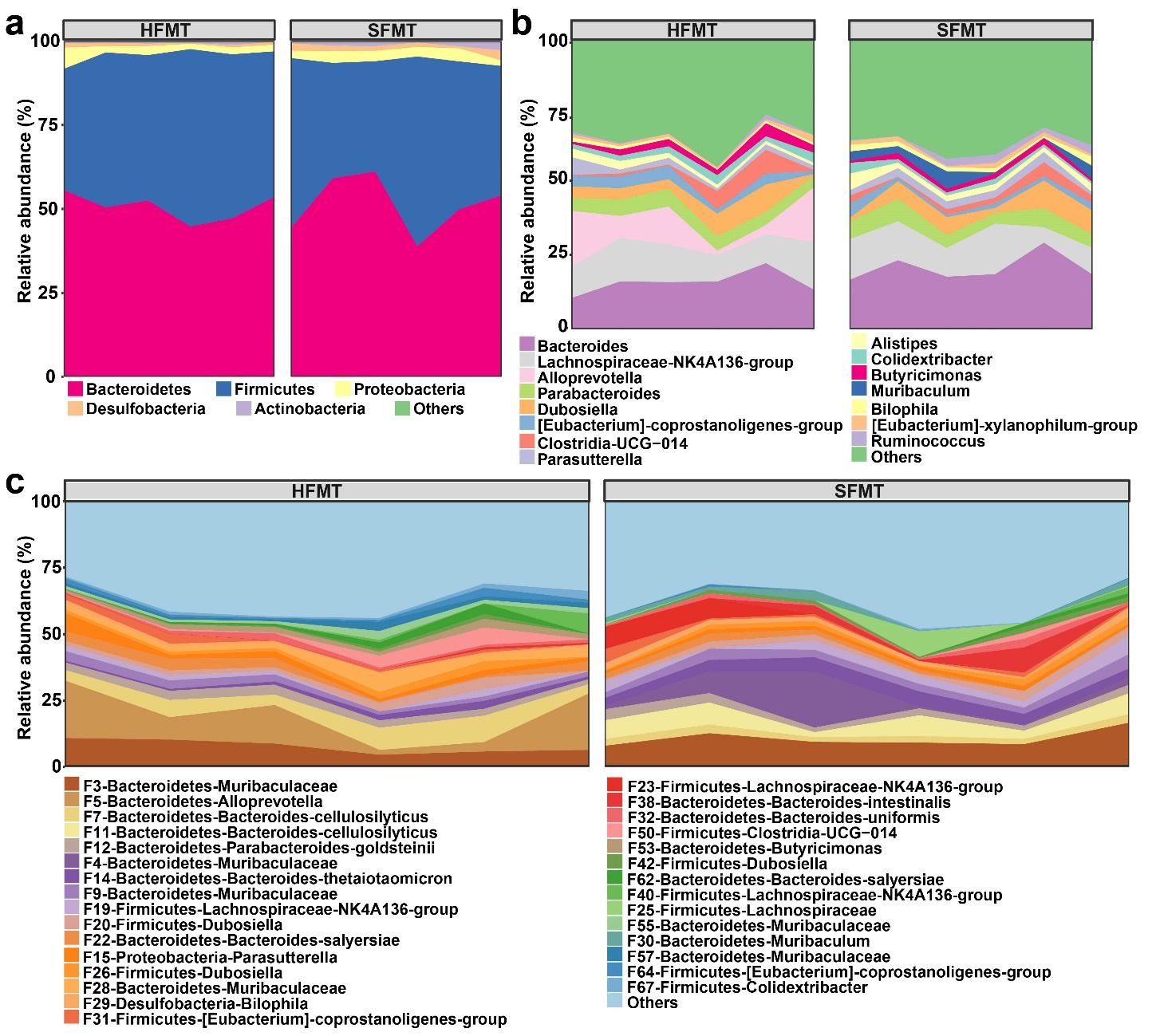


Supplementary Figure 8. Overall gut microbiota composition of recipients with SFMT differed from those with HFMT. (a) Relative abundances of the top 5 phyla. (b) Relative abundances of the top 15 genera. (c) Relative abundances of the top 30 features. n = 6 per group. These feature IDs in mice were obtained from the new analysis process and are therefore different from those of human datasets metioned before.


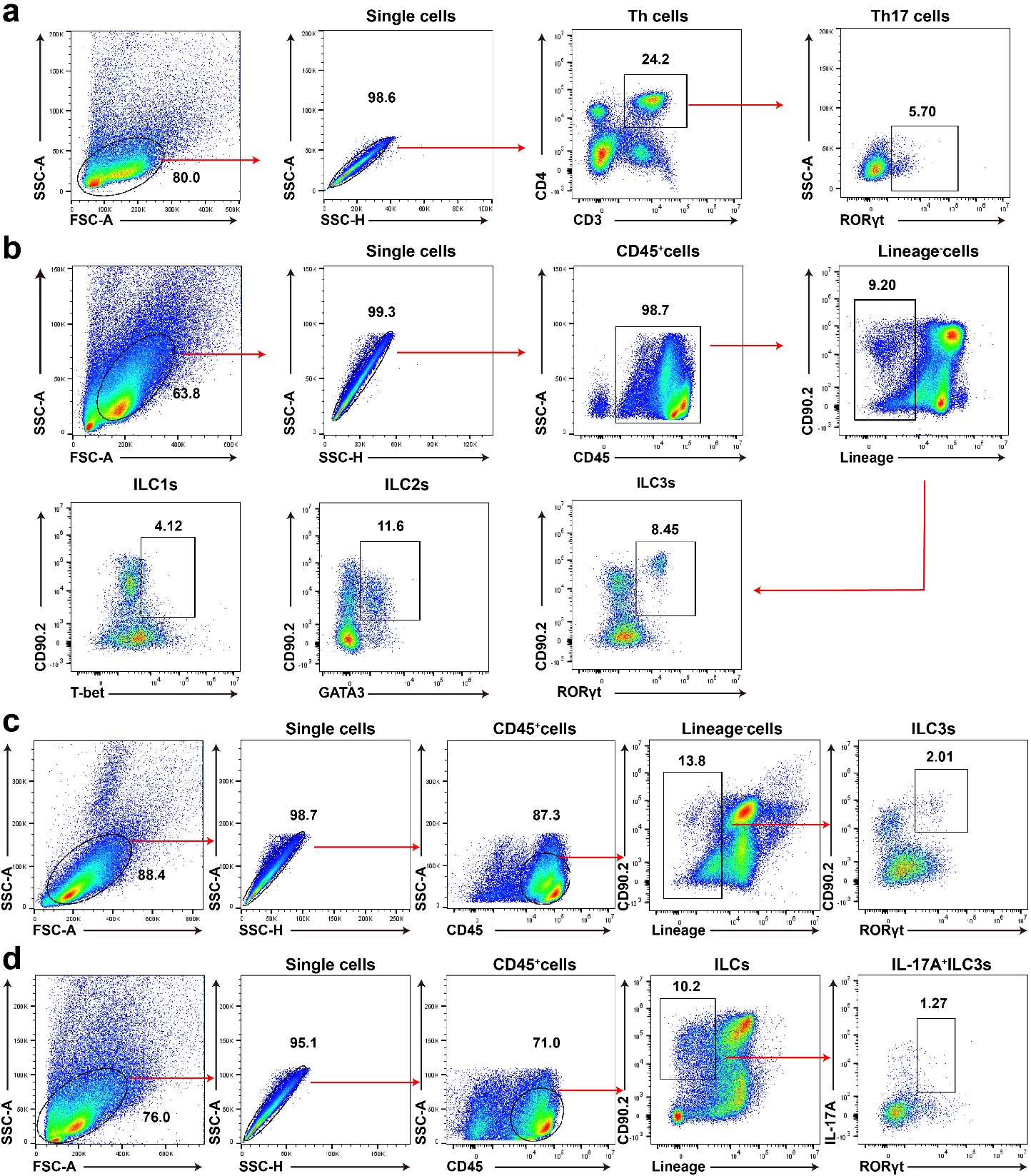


Supplementary Figure 9. Gating strategies for flow cytometry analysis. (a) Gating strategy for the analysis of RORγt^+^Th17 cells in the FMT trial. (b) Gating strategy for the analysis of total ILC1s, ILC2s, and ILC3s in the FMT trial. (c) Gating strategy for the analysis of total ILC3s in the 12-KLCA trial. (d) Gating strategy for the analyse of IL17A^+^ILC3s in the 12-KLCA trial.
