# Supplementary material for: Gut microbiota-derived 12-ketolithocholic acid suppresses the IL-17A secretion from colonic group 3 innate lymphoid cells to prevent the acute exacerbation of ulcerative colitis
Source: Gut Microbes. 2023 Dec 8;15(2):2290315. doi: 10.1080/19490976.2023.2290315 (PMC10730201; doi:10.1080/19490976.2023.2290315)
Supplement: Supplementary methods_20231122.docx [file KGMI_A_2290315_SM3540.docx]

## Supplementary Methods

## *Medical institutes that enrolled clinical participants*

Jiangsu Hospital of Chinese Medicine (Nanjing, Jiangsu, China); Chongqing Hospital of Chinese Medicine (Chongqing, China); Guangdong Hospital of Chinese Medicine (Guangzhou, Guangdong, China); Dong Fang Hospital of Beijing University of Chinese Medicine (Beijing, China); The First Affiliated Hospital of Henan University of Chinese Medicine (Zhengzhou, Henan, China); The First Affiliated Hospital of Tianjin University of Chinese Medicine (Tianjin, China); Yueyang Hospital of Integrated Traditional Chinese and Western Medicine of Shanghai University of Traditional Chinese Medicine (Shanghai, China); Shanxi Hospital of Chinese Medicine (Taiyuan, Shanxi, China); The First Affiliated Hospital of Shandong University of Chinese Medicine (Jinan, Shandong, China); Shanghai Tenth People’s Hospital (Shanghai, China); Tianjin People’s Hospital (Tianjin, China).

## *Forty synthetic standards of bile acid targeted quantification*

Conjugated primary bile acids: glycocholic acid (GCA), glycochenodeoxycholic acid (GCDCA), glycoursodeoxycholic acid (GUDCA), taurocholic acid (TCA), taurochenodesoxycholic acid (TCDCA), tauroursodeoxycholic acid (TUDCA), tauro-α-muricholic acid (TαMCA), tauro-β-muricholic acid (TβMCA).

Deconjugated primary bile acids: cholic acid (CA), chenodeoxycholic acid (CDCA), ursodeoxycholic acid (UDCA), α-muricholic acid (α-MCA), β-muricholic acid (β-MCA), 3β-cholic acid (3β-CA), 3β-ursodeoxycholic acid (3β-UDCA), 23-norcholic acid (NCA).

Secondary bile acids: deoxycholic acid (DCA), lithocholic acid (LCA), isolithocholic acid (Iso-LCA), epiallolithocholic acid (EALCA), 7-ketolithocholic acid (7-KLCA), 12-ketolithocholic acid (12-KLCA), murideoxycholic acid (MDCA), ursocholic acid (UCA), hyocholic acid (HCA), glycodeoxycholic acid (GDCA), taurodeoxycholic acid (TDCA), glycolithocholic acid (GLCA), lithocholyltaurine (TLCA), glycoursocholanic acid (GUCA), glycohyocholic acid (GCHCA), taurohyocholic acid (THCA), dehydrocholic acid (DHCA), hyodeoxycholic acid (HDCA), glycohyodeoxycholic acid (GHDCA), taurohyodeoxycholic acid (THDCA), 6,7-diketolithocholic acid (6,7-DLCA), 7,12-diketolithocholic acid (7,12-DKLCA), allocholic acid (ALCA), nor-desoxycholic acid (Nor-DCA).
